# TF-High-Evolutionary: In Vivo Mutagenesis of Gene Regulatory Networks for the Study of the Genetics and Evolution of the *Drosophila* Regulatory Genome

**DOI:** 10.1093/molbev/msae167

**Published:** 2024-08-09

**Authors:** Xueying C Li, Vani Srinivasan, Ian Laiker, Natalia Misunou, Nicolás Frankel, Luisa F Pallares, Justin Crocker

**Affiliations:** European Molecular Biology Laboratory, Heidelberg, Germany; European Molecular Biology Laboratory, Heidelberg, Germany; Instituto de Fisiología, Biología Molecular y Neurociencias (IFIBYNE), Consejo Nacional de Investigaciones Científicas y Técnicas (CONICET) y Universidad de Buenos Aires (UBA), Buenos Aires 1428, Argentina; European Molecular Biology Laboratory, Heidelberg, Germany; Instituto de Fisiología, Biología Molecular y Neurociencias (IFIBYNE), Consejo Nacional de Investigaciones Científicas y Técnicas (CONICET) y Universidad de Buenos Aires (UBA), Buenos Aires 1428, Argentina; Friedrich Miescher Laboratory, Max Planck Society, Tübingen, Germany; European Molecular Biology Laboratory, Heidelberg, Germany

**Keywords:** mutagenesis, experimental evolution, transcription factor, *Drosophila*, *cis*-regulatory evolution, evo devo

## Abstract

Understanding the evolutionary potential of mutations in gene regulatory networks is essential to furthering the study of evolution and development. However, in multicellular systems, genetic manipulation of regulatory networks in a targeted and high-throughput way remains challenging. In this study, we designed TF-High-Evolutionary (HighEvo), a transcription factor (TF) fused with a base editor (activation-induced deaminase), to continuously induce germline mutations at TF-binding sites across regulatory networks in *Drosophila*. Populations of flies expressing TF-HighEvo in their germlines accumulated mutations at rates an order of magnitude higher than natural populations. Importantly, these mutations accumulated around the targeted TF-binding sites across the genome, leading to distinct morphological phenotypes consistent with the developmental roles of the tagged TFs. As such, this TF-HighEvo method allows the interrogation of the mutational space of gene regulatory networks at scale and can serve as a powerful reagent for experimental evolution and genetic screens focused on the regulatory genome.

Gene regulation plays a critical role in development and evolution (Carroll 2000; Yanai 2018). Transcription factors (TFs) and their target genes establish complex networks that define the spatial and temporal patterns of gene expression (Fig. 1a). However, how regulatory information is encoded in these networks and how the networks can change over evolutionary timescale remain open questions. In recent years, mutational scans have provided important insights into the evolvability of regulatory elements (Metzger et al. 2015; Fuqua et al. 2020; Galupa et al. 2023; Li et al. 2023), but expanding this approach to a network level requires multiplexed, high-throughput genetic perturbations, a technically challenging goal in nonmicrobial systems. Laboratory evolution allows close examination of network dynamics in a short evolutionary timescale. But in multicellular organisms, given their low mutation rate and small experimental population sizes, this experimental approach can only explore standing genetic variation and not de novo mutations (Schlötterer 2023), preventing interrogation of broader sequence spaces. Additionally, mapping causal variants, which can be broadly distributed with low-effect sizes, remains a challenge (Schaid et al. 2018). As international efforts toward understanding the effects of perturbations in human and model systems increase (Boyle et al. 2017; Conery and Grant 2023), new, high-throughput perturbation approaches are essential.

**Fig. 1.**
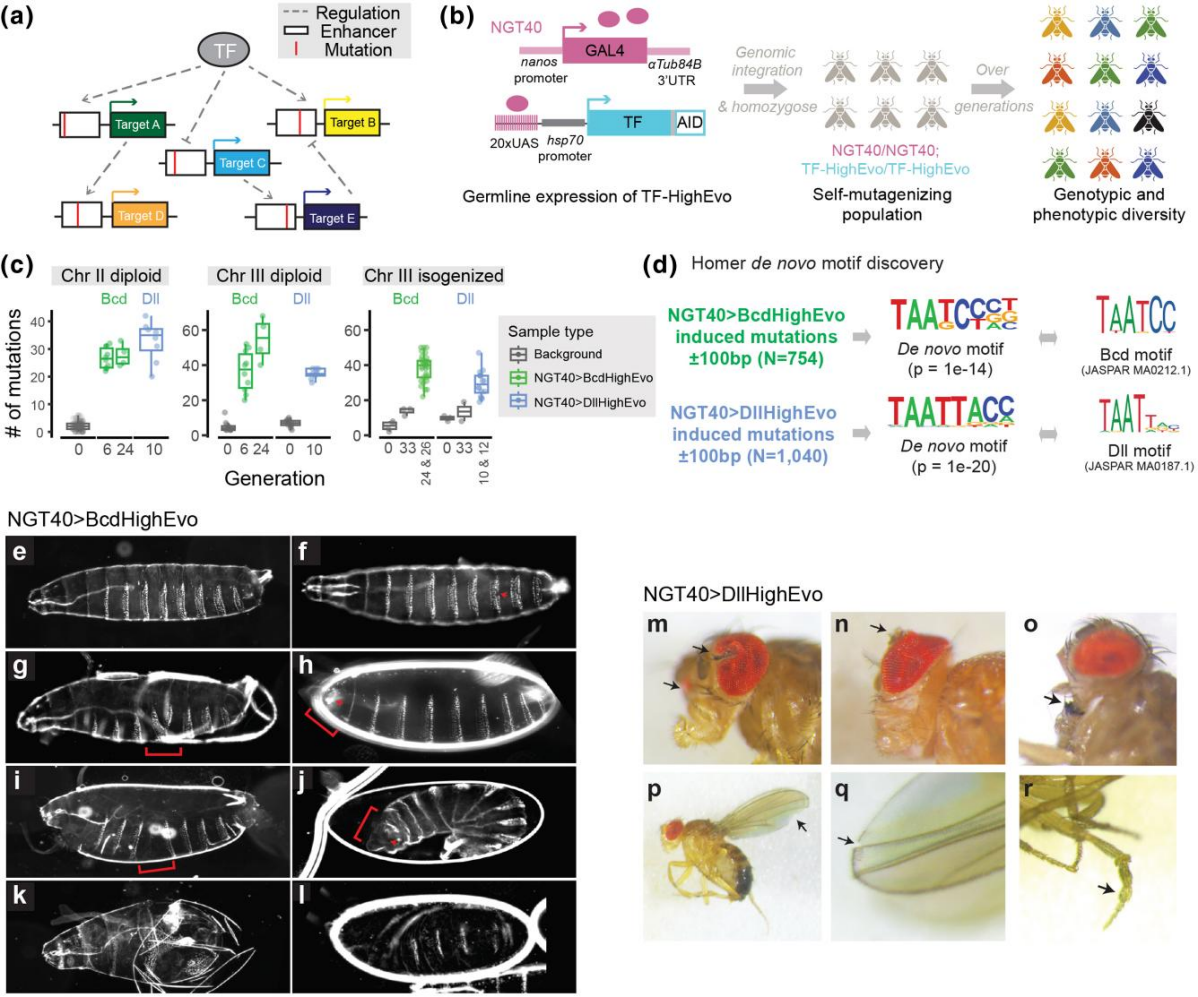
TF-HighEvo-induced mutations in vivo in a TF-specific manner. a) A TF regulates the expression of its target genes by binding to enhancers (white boxes), together constituting a complex network, often with cross-regulation (dashed lines). Mutagenesis (vertical lines) across multiple enhancers is expected to deepen our understanding of the evolvability of the network. b) Experimental design of TF-HighEvo mutagenesis. c) Number of mutations in mutagenized and background samples. Diploid data correspond to individuals sampled from the self-mutagenizing populations. Isogenic data correspond to chr3-isogenic lines established from single male individuals. G0 data correspond to interindividual variation in NGT40 (chr II) and BcdHighEvo or DllHighEvo background stocks (chr III). G33 data correspond to mutation accumulation at 29 °C for 33 generations without NGT40. Data from Generations 24 and 26 were pooled for BcdHighEvo, and data from Generations 10 and 12 were pooled for DllHighEvo. d) De novo motif discovery around BcdHighEvo- and DllHighEvo-induced mutations (C-T and G-A changes). *N* denotes the number of sequences scanned. e to l) Cuticle phenotypes in an NGT40>BcdHighEvo population at the fourth generation of mutagenesis. e) Normal cuticle. f to l) Mutant phenotypes, with specific defects indicated by red arrows or brackets; see the main text for description. m to r) Defects in adult morphology in an NGT40>DllHighEvo/TM2 population. m and n) Eye abnormalities. o) Ectopic bristles in mouth parts. p and q) Wing defects. r) Outgrowth in legs.

Here, we developed a strategy to mutagenize transcriptional targets genome-wide across gene regulatory networks in vivo by fusing a TF to an activation-induced deaminase (AID), which induces C-to-T or G-to-A mutations at the binding sites (Yang et al. 2016). We named this design TF-High-Evolutionary (TF-HighEvo) after the fictional character High Evolutionary, for its potential in evolving new life forms (DeFalco et al. 2019). To establish and validate the mutagenesis strategy, we tagged the classical TFs Bicoid (Bcd) and Distal-less (Dll). These factors are essential for fly development with important roles in early embryogenesis (Driever and Nüsslein-Volhard 1988) and appendage growth (Carroll et al. 1994), respectively. The fused protein (referred to as TF-HighEvo hereafter) was placed downstream of 20 upstream activating sequence (UAS) motifs and a core promoter (Fig. 1b; see supplementary data file S1, Supplementary Material online for the construct map), which can be activated by a germline-specific Gal4 driver, namely, NGT40. The NGT40 driver is controlled by a *nanos* promoter and the 3′UTR of αTub84B (Tracey et al. 2000; Fig. 1b), with known activity in oocytes (Tracey et al. 2000) and testis (White-Cooper 2012). By combining NGT40 and TF-HighEvo (supplementary fig. S1, Supplementary Material online), we generated “self-mutagenizing” *Drosophila* populations that continuously accumulated heritable mutations around their associated TF-binding sites. Over time, the self-mutagenizing populations are expected to exhibit genotypic and phenotypic diversity across the hundreds to thousands of targets of their corresponding TFs across the genome (Ochoa-Espinosa et al. 2009; Brennan et al. 2023; Fig. 1b).

To characterize the efficacy of mutagenesis, we sequenced the genome of 45 individual flies sampled along the time course of TF-HighEvo mutagenesis (see supplementary fig. S2a, Supplementary Material online for the full sampling scheme), 34 individuals from background stocks, 71 chr3-isogenic lines derived from mutagenized individuals (see supplementary fig. S2b, Supplementary Material online for the isogenizing crosses), as well as balancer stocks used in the crosses (see Methods). Using a customized computational pipeline, we identified private genetic variants that were only present in mutagenized or nonmutagenized individuals (from now on “background set”), while allowing for variants shared among individuals within each set. These variants are most likely to be de novo mutations caused by TF-HighEvo mutagenesis or reflect the baseline mutation rate in the case of the background samples. We focused on chr2 and chr3, where we could confidently account for background genetic variation (see Methods, supplementary fig. S3, Supplementary Material online for technical details). Among the 1,754 private variants identified in the individual samples, 93.4% of the variants were single-nucleotide polymorphisms (SNPs), 3.8% insertions and deletions, and 2% multinucleotide polymorphisms (supplementary fig. S4a, Supplementary Material online). The characteristics of the identified private variants were consistent with the stochastic nature and recent appearance of de novo mutations: 65.6% were found only in one individual (“singletons”; supplementary fig. S4a, Supplementary Material online); out of 4,078 occurrences of the 1,754 private variants, 88.3% were heterozygous.

To characterize the mutation rate associated with our approach, we compared the de novo mutations induced by NGT40>TF-HighEvo with the following: (i) the level of interindividual variation caused by naturally occurring de novo mutations, reflected by the number of private variants identified in randomly sampled individuals in background stocks (G0 in Fig. 1c); and (ii) mutations accumulated in TF-HighEvo stocks over 33 generations at 29 °C in the absence of NGT40. We found that mutations rapidly accumulated in TF-HighEvo-mutagenized individuals, with a mean of 20 to 40 mutations in each chromosome after 6 to 10 generations compared with 2 to 7 mutations in the background flies (Fig. 1c, left and middle panels). For BcdHighEvo-induced mutagenesis where two time points were sampled, the mean number of mutations in chr3 increased from 36.5 at generation 6 to 54.75 at generation 24, consistent with continuous mutation accumulation. In chr3-isogenic lines derived from males of the TF-HighEvo populations, we found an average of 38.4 mutations in the isogenized chr3 derived from Generation 24 and 26 of BcdHighEvo mutagenesis, and 29.4 mutations derived from Generation 10 of DllHighEvo mutagenesis (Fig. 1c, right panel**)**, both higher than G0 (a mean of 5.25 for BcdHighEvo and 9.7 for DllHighEvo). To evaluate the necessity of the germline driver as well as to characterize the baseline mutagenic activity of TF-HighEvo under the *hsp70* promoter, which has never been tested in *Drosophila* before, we kept TF-HighEvo stocks without the germline driver at 29 °C for 33 generations and sequenced the isogenic derivatives of them. We found an average of 13.6 mutations in the third chromosome after 33 generations, consistent with a much slower mutation-accumulation rate than the NGT40-driven TF-HighEvo populations (Fig. 1c, right panel). These results indicate that, consistent with the previously reported AID-domain’s ability to generate targeted mutations at a single locus (Yang et al. 2016), our approach increases mutation frequencies genome-wide.

Using the data from the isogenic lines and normalizing to the number of bases covered in chr3 (see Methods), we estimated a mutation rate of 4.7 ± 0.5 × 10^−8^ per base per generation per diploid genome in an NGT40>BcdHighEvo population, and 8.1 ± 1.3 × 10^−8^ in an NGT40>DllHighEvo population. This estimate is one order of magnitude higher than the endogenous mutation rate in *Drosophila* (8.4 × 10^−9^, 95% confidence interval 3.6 × 10^−9^ to 1.6 × 10^−8^; Haag-Liautard et al. 2007). Importantly, we note that the extrapolated per-base mutation rate is an underestimate of the efficiency of TF-HighEvo mutagenesis, as these mutations are not distributed randomly in the genome but biased toward the TF-associated regulatory regions.

We next studied the genomic features associated with the identified mutations to understand whether they were enriched in regulatory regions with TF-specific activity. This dataset includes a total of 2,704 variants identified in individual samples (1,754) and isogenic lines (950) (supplementary data file S2, Supplementary Material online). First, consistent with AID's role as deaminase, a large majority of the mutations in mutagenized samples were C-to-T and G-to-A mutations (1,883/2,329, 80.9%), a level significantly higher than background variation (91/201, 45.3%, Fisher's exact test, *P* < 2.2e−16, odds ratio = 5.10) (supplementary fig. S3c, Supplementary Material online). Most of the mutations were distributed in introns (39.1%) and exons (24.2%), not significantly different from the background distribution (supplementary fig. S3d, Supplementary Material online).

To test whether the mutations were enriched around TF-binding sites, we scanned 200 bp–long sequences centered around C-to-T and G-to-A mutations in BcdHighEvo- or DllHighEvo-mutagenized samples with HOMER for de novo motif discovery. The only motif significantly enriched in the BcdHighEvo and DllHighEvo input sequences (Fig. 1d, middle panel) corresponded to the canonical motif for Bcd (JASPAR MA0212.1, *P* = 1e−14) and Dll (JASPAR MA0187.1, *P* = 1e−20), respectively (Fig. 1d, right panel). We further found that the Bcd/DllHighEvo-induced mutations were more likely to be within 100 bp distance to Bcd/Dll motif clusters and ChIP-seq peaks than randomly sampled natural variants in the *Drosophila* Genetic Reference Panel (DGRP; supplementary fig. S4e, Supplementary Material online). Both sets of mutations were enriched for Bcd and Dll ChIP-seq peaks, suggesting general regulatory activity, but the odds ratio of Bcd ChIP-seq enrichment was consistently higher for BcdHighEvo-associated sequences than DllHighEvo-associated or background sequences (supplementary fig. S4e, Supplementary Material online) and vice versa. Additionally, the TF-HighEvo-induced mutations were enriched around ATAC-seq peaks in testis (supplementary fig. S4e, Supplementary Material online), suggesting that, in some cases, the efficacy of TF-HighEvo mutagenesis might depend on the chromatin accessibility in germ cells. Together, these results indicate that TF-HighEvo-induced mutations are highly TF specific and their location in the genome tightly matches the known regulatory sequences associated with the tagged TF.

Given that the genomic analyses confirmed that TF-HighEvo mutagenesis was happening in the targeted regulatory networks, we expected that some of the mutations recapitulate known phenotypic variation associated with network perturbations. Bicoid is a TF that forms a concentration gradient along the anterior–posterior axis in early embryos, governing the downstream segmentation network (Driever and Nüsslein-Volhard 1988). Consistent with this role, we found a variety of segmentation defects in larval cuticles in the BcdHighEvo population after four generations of mutagenesis (Fig. 1e to l), ranging from mild defects such as missing trichomes (Fig. 1f) to severe defects such as segment fusion (Fig. 1g), failure to form anterior structures (no mouth hooks, Fig. 1h), segment expansion (Fig. 1i), no head involution (Fig. 1j), almost-only anterior segments (Fig. 1k), and skipped segments (Fig. 1l). The severe defects were found in BcdHighEvo populations at a rate of 2% but never in wild-type stocks. In the chr3-isogenic lines derived from BcdHighEvo mutagenesis, we found at least four lines (out of 29) consistently showing severe cuticle defects (supplementary fig. S5a to e, Supplementary Material online), some associated with loss of genitals in the adult stage (supplementary fig. S5f, Supplementary Material online). Additionally, around 20% of the lines were sterile or lethal when chr3 was homozygosed, with the lethal effects distributed in different developmental stages (supplementary fig. S5g to i, Supplementary Material online). Together, this phenotypic diversity paralleled “The Heidelberg Screen” (Nüsslein-Volhard and Wieschaus 1980) that uncovered multiple targets of Bcd across the embryonic segmentation network, demonstrating the diverse yet focused phenotypic space that TF-HighEvo mutagenesis allows to interrogate.

Flies from the DllHighEvo-mutagenesis populations had abnormalities consistent with the broad roles of Dll across development (Panganiban and Rubenstein 2002), including eyes, mouth parts, wings, and legs (Fig. 1m to r; also see supplementary text and fig. S1, Supplementary Material online for details). Crosses with the eye mutants showed that the phenotypes were heritable with a complex genetic basis (supplementary text and fig. S6, Supplementary Material online), demonstrating the potential of this approach for studying polygenic traits. To complement the morphological assays, we assayed the crawling behavior of third-instar larvae and found that two lines derived from DllHighEvo mutagenesis showed aberrant “wiggly” movements when crawling forward (supplementary videos S1 and S2, Supplementary Material online), consistent with Dll's role in the development of the nervous system (Panganiban and Rubenstein 2002). Together, the wide range of phenotypes found in the TF-HighEvo populations demonstrates that this approach allows a “mutational scan” at the network level, with a bias toward TF-binding regions, providing a powerful reagent for future experimental evolution studies and genetic screens.

In summary, we show that our in vivo TF-HighEvo approach (i) generates a large number of mutations recurrently across generations, (ii) at a rate of one order of magnitude higher than the *Drosophila* mutation rate, (iii) such mutations are concentrated around TF-binding sites and regions with regulatory activity, and (4) generate phenotypic variation in line with the known function of the targeted regulatory network. Accordingly, the in vivo TF-HighEvo mutagenesis approach is expected to have a number of applications in the fields of genetics and evolution. First, the self-mutagenizing populations provide ideal materials for studying network evolvability with experimental evolution. With an increased mutation rate and a mutational bias toward associated regulatory regions (Fig. 1), the TF-HighEvo-expressing populations might respond differently from wild-type populations to directed selection, with a higher likelihood to sample adaptive mutations from the regulatory sequences in the targeted networks. Although transcriptional networks can consist of tens of thousands of regulatory elements, their target size for mutagenesis is still small compared to the whole genome. For example, Brennan et al. (2023) identified 37,556 *cis*-regulatory elements bound by important TFs in *Drosophila* embryos, together spanning 9.1 Mb, which takes up only 7.6% of the fly euchromatin genome. Therefore, experimental evolution based on de novo or chemically induced random mutations might not be able to fully sample these mutations within a short period of time (Li et al. 2024). The TF-HighEvo method is expected to “speed up” experimental evolution primarily using mutations in the targeted gene regulatory network.

As a new mutagenesis method, our approach has a few strengths compared to chemical mutagenesis, hybrid dysgenesis, and CRISPR-based methods. Compared to chemical mutagenesis, mutations are “automatically” generated by TF-HighEvo once the mutagenic constructs are integrated into the genome, removing the need to handle dangerous chemicals such as ethyl methanesulfonate. The self-mutagenizing populations can be left in the lab for years to accumulate mutations, making it an ideal “background project.” Although P-element mutagenesis provides a similar level of convenience in experimental procedures, the TF-HighEvo approach generates point mutations that are less likely to be disruptive than P-element insertions. Therefore, it may be more suited than hybrid dysgenesis for understanding effects of point mutations that underlie mild phenotypes. Compared to the CRISPR-based method, our method does not require designing site-specific guide RNAs, which often relies on prior knowledge of TF-binding motifs that are biased toward high-affinity binding sites. In fact, we expect the in vivo TF-HighEvo mutagenesis to provide orthogonal evidence to previous knowledge of TF-binding activity, leading to the discovery of novel *cis*-regulatory elements. In the future, this technology can produce large, systematically collected datasets focused on the effects of perturbations across regulatory networks, which can be used to improve models of gene regulation (Arnold et al. 2013; Li et al. 2022).

There are a few factors to keep in mind when designing future TF-HighEvo experiments (see supplementary text, Supplementary Material online for more technical considerations). It is possible that this approach might not work with all TFs. In addition to Bcd and Dll, we tested the AID fusion with Eyeless, a TF critical for eye development (Clements et al. 2008), and Gal4, a yeast-derived TF commonly used in *Drosophila* genetics. The two lines did not exhibit obvious morphological phenotypes, so we did not follow-up on them with whole-genome sequencing. Therefore, it remained inconclusive if these two fusions were mutagenic. It is possible that fusing AID to TFs might be detrimental to the DNA-binding activity of certain TFs. Also, some TFs are more sensitive to chromatinized DNA (Isbel et al. 2022), which may limit their ability to mutagenize regulatory elements. Additionally, germline expression of TF-HighEvo is essential for generating heritable mutations in the present design, but it is important to keep in mind that mutations might not occur if the TF targets were inaccessible in the germline. Lastly, the strategy of TF-HighEvo mutagenesis over generations might be of limited use if the main interest is to isolate lethal or highly deleterious mutations, which would be selected against over time.

Our approach offers an innovative strategy that, for the first time, allows a large-scale assessment of the effect of de novo mutations during the experimental evolution of multicellular organisms. In the future, the approach may be combined with other genome-editing and genomics technologies, such as the use of engineered base editors (Xiong et al. 2023) or strategies for inducing somatic expression (Liu et al. 2021). The approach can also be applied in different organisms, to address diverse biological questions ranging from evolutionary genetics and developmental biology to synthetic and system biology.

## Methods

### Fly Genetics

TF-HighEvo constructs were designed according to Yang et al. (2016). Coding sequence of Bicoid (FBpp0081165) or Distal-less (FBpp0072286) with the stop codon removed was fused with an 8-amino-acids-long linker and AID*^ΔNES^* at its 3′ end as described in Yang et al. (2016). The constructs were synthesized and cloned into pJC-TAL-1XH vector (Crocker et al. 2016) by Genscript, downstream of 20× UAS-binding sites and a *hsp70* promoter, yielding plasmid pXL-Bcd-AID-delN and pXL-Dll-AID-delN (see supplementary data file S1, Supplementary Material online for sequence maps). The plasmids were integrated into the fly genome at the VK33 landing site via PhiC31 integrase with the injection service provided by GenetiVision.

To start the in vivo mutagenesis, TF-HighEvo constructs were crossed with NGT40 (BDSC #4442, y[1] w[*]; P{w[+mC] = GAL4-nanos.NGT}40) under the *crossing scheme* in supplementary fig. S1, Supplementary Material online. The first flies with a homozygous genotype of ; NGT40/NGT40; TF-HighEvo/TF-HighEvo were considered G0, when the mutagenesis began. The self-mutagenizing populations were expanded and kept in bottles with a population size of 200 to 500 at 25 °C under standard fly-rearing conditions. They were flipped every ∼16 d, with overlapping generations allowed. Over the years 2021 to 2023, we started three rounds of mutagenesis, designated as “XL1,” “VS2,” and “XL3,” respectively. During the time course of mutagenesis, we sampled the populations for genome sequencing and phenotypic analysis at different time points, ranging from the 4th to the 26th generations (supplementary fig. S2a, Supplementary Material online). Of note, some flies from Round XL1 carried the TM2 balancer, which allowed us to explore the effects of balancers on mutation accumulation (see supplementary text, Supplementary Material online). In addition, homozygous stocks of TF-HighEvo were placed at 29 °C over generations to characterize the background rate of TF-HighEvo mutagenesis without the NGT40 driver.

At Generations 24 to 26 of BcdHighEvo mutagenesis (Round XL1) and Generations 10 to 12 of DllHighEvo mutagenesis (Round VS2), we outcrossed 20 to 30 mutagenized male individuals to a third-chromosome balancer (; ;Sb/TM6B) to establish chr3-isogenic lines, following the crossing scheme in supplementary fig. S2b, Supplementary Material online. We also established isogenic lines following the same procedure from Generations 2 and 5 of BcdHighEvo and DllHighEvo mutagenesis (Round XL3) for phenotypic analyses (supplementary fig. S5g, Supplementary Material online), as well as from nonmutagenized TF-HighEvo stocks. Of note, the isogenizing crosses allowed us to end the mutagenesis, because we could select against the NGT40 chromosome based on the red eye color (the TF-HighEvo constructs also carried a *w^+^* marker, but they only conferred yellow eye color when integrated at the VK33 landing site). This caused a slight difference between the crosses derived from the mutagenizing populations and the control stocks. In the latter case, we could not specifically select chr2 from the balancer stock (indicated by the white color in supplementary fig. S2b, Supplementary Material online) due to the lack of markers, resulting in a different genetic background for this chromosome. This difference should be considered when interpreting the phenotypic results.

At Generation 4 of DllHighEvo mutagenesis (Round XL1), we observed a number of flies with eye defects. We individually outcrossed male flies with such eye defects to *w*^1118^ virgins to establish inbred lines, referred to as “eye lines” hereafter (supplementary fig. S6b and text, Supplementary Material online). The F1 flies did not show eye phenotypes. From F2 onward, we manually selected flies with eye defects every generation for over 20 generations. At the end of the selection, we generated 6 lines (Eye1, Eye2, Eye3-0, Eye3-1, Eye3-2, and Eye4) stably expressing the eye phenotype at a rate ranging from 30% to over 90% at 25 °C. The six lines were derived from four founder males, with the shared paternal ancestry reflected in their labels, i.e. Eye3-0, Eye3-1, and Eye3-2 were all derived from Male #3.

For each of the eye lines, we established multiple chr3-isogenic lines from 2 to 3 males sampled from each line following the crossing scheme in supplementary fig. S6c, Supplementary Material online. In particular, some flies in Eye1, Eye3, and Eye3-1 had red eyes because of the presence of NGT40 (other lines had white eyes, having lost NGT40 and DllHighEvo during the artificial selection). The eye color can, therefore, be a marker for the presence of paternal chr2 in the isogenizing crosses. For these three lines, we established chr3-isogenic lines from F2 flies with and without red eyes, to study the contribution of variants on chr2 to the eye phenotype (supplementary fig. S6c, Supplementary Material online). The eye lines were sequenced at the time when they showed stable expression of phenotypes (roughly after 15 generations of artificial selection). For each eye line, two isogenic descendants that showed the highest frequency of eye defects at 29 °C were sequenced.

### Genome Sequencing

We sequenced the following: (i) individual flies from the self-mutagenizing populations, sampled across generations and populations; (ii) all the background stocks, including TF-HighEvo stocks, NGT40, all balancers, and *w*^1118^; (iii) the chr3-isogenic lines, with 2 flies per isogenic lines sequenced, as replicates; (iv) flies from eye lines (pooling 6 to 12 flies per line) and their chr3-isogenic descendants (see supplementary table S3, Supplementary Material online for a comprehensive list).

Genomic DNA was extracted with a Qiagen DNeasy Tissue Kit. The DNA was tagmented with a customized Tn5 protocol and sequenced in 75 or 110 bp paired-end on an Illumina NextSeq 500 or NextSeq 2000 at EMBL GeneCore. The sequences were deposited at ArrayExpress (EMBL-EBI) under experiment no. E-MTAB-13796.

### Read Mapping and Variant Calling

The reads were mapped to the dm6 genome with Bowtie2 (Langmead and Salzberg 2012). The duplicated reads were removed with Picard tools (Anon 2019). There were on average 26 million reads per sample after removing the duplicates, equivalent to an average sequencing depth of 19.2.

The variants were detected by a joint freebayes (Garrison and Marth 2012) call, including all 273 samples, with requirements of a minimal coverage of 10, a minimal mapping quality of 30, and a minimal base quality of 20. This resulted in ∼4.8 million variant calls in chrX, chr2, and chr3. As a preliminary quality filter, variants with a QUAL score <10 were removed, leaving ∼1.5 million variants in the dataset. The variants were annotated with ANNOVAR (Wang et al. 2010).

### Identification of De Novo Mutations

An overview of the computational pipeline to identify the de novo mutations in the mutagenized and control samples is shown in supplementary fig. S3, Supplementary Material online. The pipeline was designed to eliminate background variation as much as possible. There were two reasons for doing this: first, although *Drosophila* lab strains were generally regarded as inbred lines with little variation among individuals, our sequencing data showed that there were pockets of heterozygosity in the background strains, suggesting genetic heterogeneity. Also, individuals in the lab strains could have accumulated de novo mutations, as expected based on a per nucleotide mutation rate of 10^−9^ (equivalent to 1.99 de novo mutations per fly per generation; Haag-Liautard et al. 2007).

The following were a few key steps in the pipeline: (i) We sequenced all the background stocks used in the crosses, which turned out to be critical for removing background variation. (ii) We treated chr2 and chr3 separately and used different background sample sets for the two chromosomes (supplementary tables S1 and S2, Supplementary Material online). This was because the two chromosomes came from different genetic backgrounds, with chr2 from NGT40 and chr3 from VK33 stocks used in the injections (supplementary fig. S1, Supplementary Material online). Also, a subset of samples was from chr3-isogenic lines, for which only the variants in chr3 were of interest. (iii) We assumed that the de novo mutations, either TF-HighEvo induced or naturally occurring, should be private to a designated subset of samples but not shared across mutagenized and background strains. We subset the data to identify private variants before further quality filters to be stringent in the criteria of private variants (i.e. we do not consider a variant to be private even if there was a low-quality call in the background samples). On an additional note, all mutations we identified in this study were alternative alleles (as opposed to the reference alleles from dm6). Although it was possible that the background strains had fixed alternative alleles and mutations at these positions could match the dm6 alleles, we did not find such cases in our study: among the 12,855 sites in chr2 and the 18,754 sites in chr3 that were homozygous for alternative alleles in the background strains, none of them was mutated (i.e. a change of genotype from 1/1 to 0/1 or 0/0).

The computational details are described as follows.

Private variants were identified with bcftools (Danecek et al. 2021) view -x -S, which selected variants that were only present in a designated set of samples in contrast to the full dataset. The full dataset included the mutagenized sample set and the background sample set, as well as a few balancers and *w*^1118^. The full dataset, mutagenized set, and background set were separately defined for chr2 and chr3 (supplementary tables S1 and S2, Supplementary Material online). For chr2, there were in total 636,143 variants prior to subsetting, using dm6 as a reference. We found 1,083 private variants across 45 mutagenized samples and 896 private variants across 123 control samples (nonmutagenized samples with NGT40 background, supplementary table S1 and fig. S3, Supplementary Material online). This is equivalent to removing 99.7% of the total variants, consistent with most variants being either fixed changes in the lab strains or standing variation shared between mutagenized and background samples. After subsetting, the private variants were filtered to remove low-quality calls, based on individual genotypes. Each sample was filtered to keep variants (i) with a sequencing depth between 10 and 200 and (ii) at least 3 reads supporting each allele (AO > 2 and RO > 2) in the case of a heterozygous call. The filtered variants were then joined across samples to merge into one dataset. To ensure a fair comparison between the mutagenized and control samples, we further removed variants that were identified as private in the mutagenized samples but were not covered in the control samples (requiring a nonmissing call in at least two samples) and vice versa. We also removed 12 samples (supplementary table S1, Supplementary Material online) that appeared to have introgression from another genetic background, indicated by a 40 kb– or a 630 kb–long high-linkage disequilibrium (LD) region in chr2L. After the filters, there were, respectively, 631 and 118 private variants across the mutagenized and control samples in chr2.

For chr3, we initially identified 26,829 private variants across 171 mutagenized samples and 728 private variants across 40 control samples, out of 621,906 variants in a full set of 227 samples (supplementary table S2 and fig. S3, Supplementary Material online). Similar to chr2, this step removed 95.6% of the total variants, suggesting that the majority of the variants in the initial call were standing variation or fixed changes. The private variants were further filtered on an individual basis as described previously, including the filters on sequencing depth and allele depth, as well as missing data. Two samples (Bcd_NM5-1a_G10 and Bcd_NM5-1b_G10) were removed owing to introgression from another genetic background.

Additionally, we took special care of the isogenic samples. In order to improve accuracy in identifying the private variants, we sequenced two flies from each isogenic line as biological replicates. In a few cases (15 lines), the male parent that was used to start the isogenic line was also sequenced. The information from a replicate sample is helpful for validating private variants in low-coverage regions. Therefore, in these cases where there were 2 to 3 replicates per sample, we kept variants that were present in more than one replicate regardless of its sequencing depth or allele depth. After adding these variants under the “relaxed” filter to the aforementioned dataset, we generated a list of 1,948 variants in chr3 that were private to the mutagenized (1,837) or control (111) samples.

There was no or very low correlation between the mean sequencing depth and the number of mutations (*r*^2^ = 0.003 and 0.03 for chr2 and chr3, respectively, supplementary fig. S4b, Supplementary Material online), and the correlation was not significant when samples with a sequencing depth <5× were removed (*P* > 0.05). In the visualization (Fig. 1) and statistics related to variant counts, all samples with a mean sequencing depth <5× were removed. Samples with a mean sequencing depth <9× but >5× were preserved in the analysis for chr3 but removed for chr2.

The mutation rate was calculated based on isogenized chr3 data, by the formula (Haag-Liautard et al. 2007): $u=N_{\mathrm{var}}/(n_{\mathrm{bp}}\times G)$, where *N*_var_ is the number of mutations, *n*_bp_ is the number of bases covered by 10 to 200 reads in each sample (generated by samtools depth, with the same quality filters as the input parameters for freebayes), and *G* is the number of generations. Because the isogenized chromosomes only harvested half of the genetic variation of the self-mutagenizing individuals, we used *n*_bp_ instead of 2*n*_bp_. In this way, *u* corresponded to the per-base mutation rate in a diploid genome. Samples with a mean depth <9× were removed in the mutation rate analysis. When the variant counts of the two replicates of an isogenic line did not match, the higher count was used. Both homozygous (87%) calls and heterozygous (13%) were counted, although the latter were usually of lower quality (e.g. low depth and possible residual background variation). The variant counts and sequencing depth were included in supplementary table S3, Supplementary Material online along with sample information.

There were additional sequence data for 36 samples from eye lines. The data were included in the joint call. We followed the same pipeline to identify private mutations in these samples, finding 3,663 variants in chr3 that were specific to the eye lines. However, most of these variants were in large LD blocks, consistent with background variation. The difficulty in removing the background variation was possibly due to the standing variation in the *w*^1118^ stock that was not captured by the limited number of individuals sequenced. Therefore, we did not further analyze these data for causal mutations underlying the eye defects.

The commands and scripts for the aforementioned pipeline are deposited at GitLab (https://git.embl.de/xuli/in-vivo-mutagenesis-of-tf-networks).

### Functional Genomics

We ran a de novo motif analysis with HOMER (Heinz et al. 2010) with the variants specific to BcdHighEvo or DllHighEvo mutagenesis. Prior to the motif analysis, we filtered the variants to keep only C-to-T and G-to-A mutations, which were 76.4% of the variants (80.9% of the SNPs). A small number of mutations were found in both BcdHighEvo and DllHighEvo samples (<5%), which were kept in both analyses. The findMotifsGenome.pl script from the HOMER suite was used to scan 200 bp–long sequences centered around either BcdHighEvo or DllHighEvo SNP. The motifs discovered were compared to known motifs in the HOMER database.

To test whether the mutations generated by TF-HighEvo were enriched for factor-specific regulatory activity, we compared our SNPs to 10,000 randomly sampled C-to-T and G-to-A SNPs in the DGRP lines (MacKay et al. 2012) to examine whether the TF-HighEvo SNPs were more likely to be within 100 bp distance of regions with regulatory activities than the DGRP SNPs. Regions with regulatory activities were defined as follows: (i) clusters of Dll motifs: we scanned the sequences around the SNPs with cluster-buster (Frith et al. 2003) to get the distance between each SNP and the nearest cluster of Dll motifs, using PWMs from the JASPAR database (Rauluseviciute et al. 2024). (ii) Bcd ChIP-seq peaks and motif clusters: as defined by Brennan et al. (2023), based on ChIP-nexus experiments with early embryos. (iii) Dll ChIP-seq peaks: the bed file was downloaded from the ChIP-ATLAS database (Zou et al. 2022) and was derived from ChIP-seq experiments in leg imaginal discs (Feng et al. 2022; GEO accession number GSE184454). (iv) Testis ATAC-seq data: obtained from Witt et al. (2021) and processed with a custom pipeline (https://github.com/laiker96/fastq_to_bam). Peaks were called with MACS2 (Zhang et al. 2008) with default parameters.

The odds ratio and *P*-values were obtained from Fisher's exact test, with the Benjamini–Hochberg correction. Each enrichment analysis was run 500 times with randomly sampled DGRP variants to generate the distribution of odds ratio and *P*-values in supplementary fig. S4e, Supplementary Material online.

A complete list of de novo mutations identified in this study and their annotations is provided in supplementary data file S2, Supplementary Material online.

### Cuticle Preparation

Flies were placed in a cage with an apple juice plate supplemented with yeast paste. They were allowed to lay eggs for an overnight period at 25 °C. The embryos were dechorionated with 50% bleach for 1.5 min and rinsed rigorously. They were then transferred to a petri dish with clean distilled water and left overnight at room temperature. On the second day, the larvae were transferred onto a glass slide and mounted in Hoyer's medium mixed with lactic acid (1:1). The slide was baked at 55 °C for 2 d before being imaged with dark field microscopy (Zeiss M2 compound microscope, 10× objective).

The cuticle images were scored based on the following criteria: severe defect—fusion, expansion, or missing segments; mild defect—missing or misaligned denticles in any segment; normal—no visible defects. For the phenotypic screening of chr3-isogenic lines, at least 30 larvae were imaged for each line. If severe defects were observed for a given slide, all larvae on this slide were imaged to get an unbiased estimate of mutant frequency.

### Larval Behavior

Overnight embryos were transferred to food bottles and placed at 25 °C for 5 d. The third-instar larvae were harvested with 10% glucose solution and placed into liquid food (yeast extract 10% m/v, glucose 10% m/v, and sucrose 7.5% m/v) overnight at 25 °C. This step helped remove the food debris from the larvae. The larvae were then placed on agar plates, where their movements were recorded using an FL3-U3-13Y3M-C CMOS camera for 1 min, at a frame rate of 30 Hz.

## Supplementary Material

msae167_Supplementary_Data

## Data Availability

The WGS reads were deposited at ArrayExpress (EMBL-EBI) under experiment E-MTAB-13796 (https://www.ebi.ac.uk/biostudies/arrayexpress/studies/E-MTAB-13796). All data supporting the findings of this study are available within the paper and its Supplementary material.
