# Supplementary material for: TF-High-Evolutionary: In Vivo Mutagenesis of Gene Regulatory Networks for the Study of the Genetics and Evolution of the Drosophila Regulatory Genome
Source: Mol Biol Evol. 2024 Aug 9;41(8):msae167. doi: 10.1093/molbev/msae167 (PMC11342961; doi:10.1093/molbev/msae167)
Supplement: msae167_Supplementary_Data [file msae167_supplementary_data.zip › 20240729_supp.pdf]

Supplementary Text

Mutagenesis at a single locus

In theory, the efficiency of TF-HighEvo mutagenesis can be measured by the mutation rate of a single binding site. In our attempt, we crossed flies of the ;NGT40/NGT40;BcdHighEvo/BcdHighEvo genotype to two synthetic enhancers "RG436" or "RG439", which contains 5x or 8x Bcd binding sites respectively (using the canonical motif "CTAATCCC"). The F1 flies have a genotype of ";NGT40/+;{BcdHighEvo}VK33/{RG436 or 439}attP2". We expected that the binding sites can be mutated by BcdHighEvo in the germ cells of these F1 flies. We individually genotyped the F2 flies by PCR and Sanger sequencing, focusing on individuals with one or two alleles of the synthetic enhancers. However, among the 66 RG436 alleles and 100 RG439 alleles genotyped, we did not find any mutations in these synthetic enhancers. Therefore, the mutation rate of a single locus might be lower than 1/166 (0.6%). Nevertheless, we note that the mutagenic efficiency tested here might be subject to the chromatin accessibility of the attP2 locus in germ cells. The mutation rate might be higher in other regions of the genome where the chromatin states are more permissive for TF binding.

Eye phenotypes in DllHighEvo mutagenesis

In the XL1 round of DllHighEvo mutagenesis where the TM2 balancer was around in the population, we found a substantial fraction of flies had abnormalities in adult eyes, including cleaves (**Fig. 1m**) or outgrowth of bristles or antenna-like structures in the eye (**Fig. 1m-n)**, among other morphological phenotypes (**Fig. 1m-r**). These phenotypes were not observed in populations without the balancer, suggesting that the TM2 balancer (carrying a ubx^{130} allele and presumably other mutations) might have provided a sensitized background. Meanwhile, sequencing data showed that the flies with visible phenotypes in the NGT40/NGT40; DllHighEvo/TM2 populations had more mutations than the populations without TM2 (**Fig. S6a**). Potentially, the presence of TM2 enabled faster accumulation of mutations by preserving homozygous-lethal mutations in the population, a well-known fact in fly genetics that can potentially be leveraged in future TF-HighEvo mutagenesis experiments.

To explore the genetic architecture of these eye phenotypes, we outcrossed four male flies (named "Eye1" to "Eye4") with the eye phenotypes individually to w^{1118} flies. The F1 offsprings did not show any phenotypes, suggesting recessive mutations. The F1 siblings were allowed to mate with each other, giving rise to F2 flies, among which a few individuals with eye phenotypes were found, suggesting that the phenotype may be caused by multiple mutations. The F2 flies with phenotypes were crossed to siblings with or without phenotypes. In the case of Eye3, three sibling crosses were established at this time, giving rise to lines Eye3-0, Eye3-1 and Eye3-2 later. From F3 onwards, we artificially selected flies

with eye abnormalities at every generation for over 20 generations, giving rise to six lines stably expressing eye phenotypes at a rate of 30%-90% at 25°C, designated as "Eye lines" hereafter (**Fig. S6b**). Interestingly, the eye defects in the stable lines differed from the founder phenotypes, including ectopic bristle growth and missing cells near the mouth parts, as well as dented eyes (**Fig. S6b**), suggesting an epistatic effect with the genetic background. After the artificial selection, lines Eye2, Eye3-2 and Eye4 had white eyes, indicating the loss of both the NGT40 and DllHighEvo constructs. During the isogenizing crosses (**Fig. S6c**), we further found that none of the lines kept the TM2 balancer or the DllHighEvo construct. Taken together, these results suggest the eye phenotypes were heritable and did not depend on the presence of the mutagenic components or TM2.

The phenotypes of the chr3-isogenic descendants further revealed a complex genetic architecture underlying the eye phenotypes (**Fig. S6d-e**). In this experiment, we generated multiple chr3-isogenic lines from the Eye lines (2-3 founder males per line, 2-3 F1 crosses per founder male) and quantified the frequency of eye phenotypes in the isogenic descendants. We found that at least one isogenic descendant per Eye line showed eye phenotypes at 25°C (**Fig. S6d**), suggesting that the chr3 of all the Eye lines carried certain causal mutations. However, the frequencies of eye defects at 25°C in the isogenic descendants were much lower than those of the stable Eye lines which they were derived from (numbers below the group labels in **Fig. S6d**), suggesting that genetic variation in chr3 only partially accounted for the phenotypes. Interestingly, the effects of chr3 from founder fly Eye3 segregated among the lines Eye3-0, Eye3-1 and Eye3-2 (**Fig. S6d**), with Eye3-2 showing a much higher level of eye defects than the other two lines, suggesting multiple mutations underlying the eye phenotype in Eye3. Due to the low percentage of eye defects at 25°C, we quantified the phenotypes at 29°C, a harsher condition that usually elevates the expression of fly morphological phenotypes. At the elevated temperature, we found high variability among the isogenic lines originated from the same Eye lines, suggesting a complex genetic architecture even within chr3.

The low frequency of eye phenotypes in the chr3-isogenic lines can be explained either by low penetrance, or by epistasis with other chromosomes. To distinguish the two possibilities, we separated "w-" and "w+" flies in the isogenizing crosses (**Fig. S6c**). The w+ flies carried red-eye markers from the NGT40 chromosome, which marked the inheritance of the chr2 of the Eye lines. This chromosome existed in the F3 flies at a 50% frequency, allowing for examination of its phenotypic effects at the population level (**Fig. S6c**). The w- flies instead inherited the chr2 from the balancer stock. By comparing w- and w+ descendants from the same Eye line, we found cases where epistasis, penetrance, or both penetrance and epistasis explained the phenotype (**Fig. S6e**). For line Eye3-1, the frequency of phenotypes was higher in w+ lines than w- lines, consistent with an epistatic effect with variants in chr2.

For line Eye3-0, the frequency of phenotypes only depended on temperature, suggesting a low penetrance of the chr3 variants and a lack of epistasis. For line Eye1, the frequency of phenotypes depended both on temperature and the presence of the paternal chr2. In summary, the genetic analysis above showed that the eye phenotypes in the DllHighEvo mutagenesis may be determined by multiple mutations across multiple chromosomes, with pervasive epistatic interactions, as well as different genetic bases in different individuals. This complexity and diversity of genetic architecture underlying a simple morphological phenotype demonstrates the great potential of network-targeted mutagenesis in revealing the genetic basis of complex traits.

Technical considerations in future TF-HighEvo experiments

From the technical perspective, we recommend considering the following factors in future applications of TF-HighEvo mutagenesis:

- The choice of TF. The mutagenic activity of TF-HighEvo may need to be tested on a factor-by-factor basis. In addition to Bcd and Dll, we tested the AID fusion with Eyeless and Gal4, but it remained to be determined if the fusion proteins worked because the two lines did not show any obvious phenotypes. Therefore, the functionality of TF-HighEvo might depend on specific TFs. For example, Bicoid was shown to have pioneer activity in early embryos (Hannon et al. 2017), which might facilitate binding and mutagenesis in germ cells.
- Mutation rate. Although we were able to elevate the mutation rate to one order of magnitude higher than the intrinsic mutation rate of *Drosophila*, the mutations accumulated were still very sparse. In the future, AID domains with higher deamination efficiency (Liu et al. 2021; Doll et al. 2023) may be used to improve the mutation rate.
- Dose of mutagenesis. In future designs, the dose of mutagenesis can be controlled by the duration (number of generations), the use of balancer chromosome, and potentially the level of expression of TF-HighEvo constructs (e.g., by temperature). Similar to EMS-mutagenesis, longer duration (a high dose) of TF-HighEvo mutagenesis would increase the probability of observing a phenotype, but the phenotypes are more likely to have complex genetic bases. Balancer chromosomes in *Drosophila* are helpful for preserving homozygous-lethal mutations, potentially accelerating mutation accumulation. Temperature or different germ-line drivers may be used to modulate the expression of TF-HighEvo, but we recommend to express TF-HighEvo at a moderate level (e.g., at 25°C) to avoid any toxicity caused by the ectopic expression.
- Start and end of mutagenesis. In this work, we controlled the start and the end of mutagenesis by crossing in and out a germline Gal4 driver. Other genetic designs could be used, e.g., placing the

TF-HighEvo directly downstream a germline promoter might make it easier to initiate but harder to end the mutagenesis.

References

Doll RM, Boutros M, Port F. 2023. A temperature-tolerant CRISPR base editor mediates highly efficient and precise gene editing in Drosophila. *Sci. Adv.* 9:eadj1568.

Hannon CE, Blythe SA, Wieschaus EF. 2017. Concentration dependent chromatin states induced by the bicoid morphogen gradient.Espinosa JM, editor. *eLife* 6:e28275.

Liu K, Deng S, Ye C, Yao Z, Wang J, Gong H, Liu L, He X. 2021. Mapping single-cell-resolution cell phylogeny reveals cell population dynamics during organ development. *Nat. Methods* 18:1506–1514.

Fig. S1.

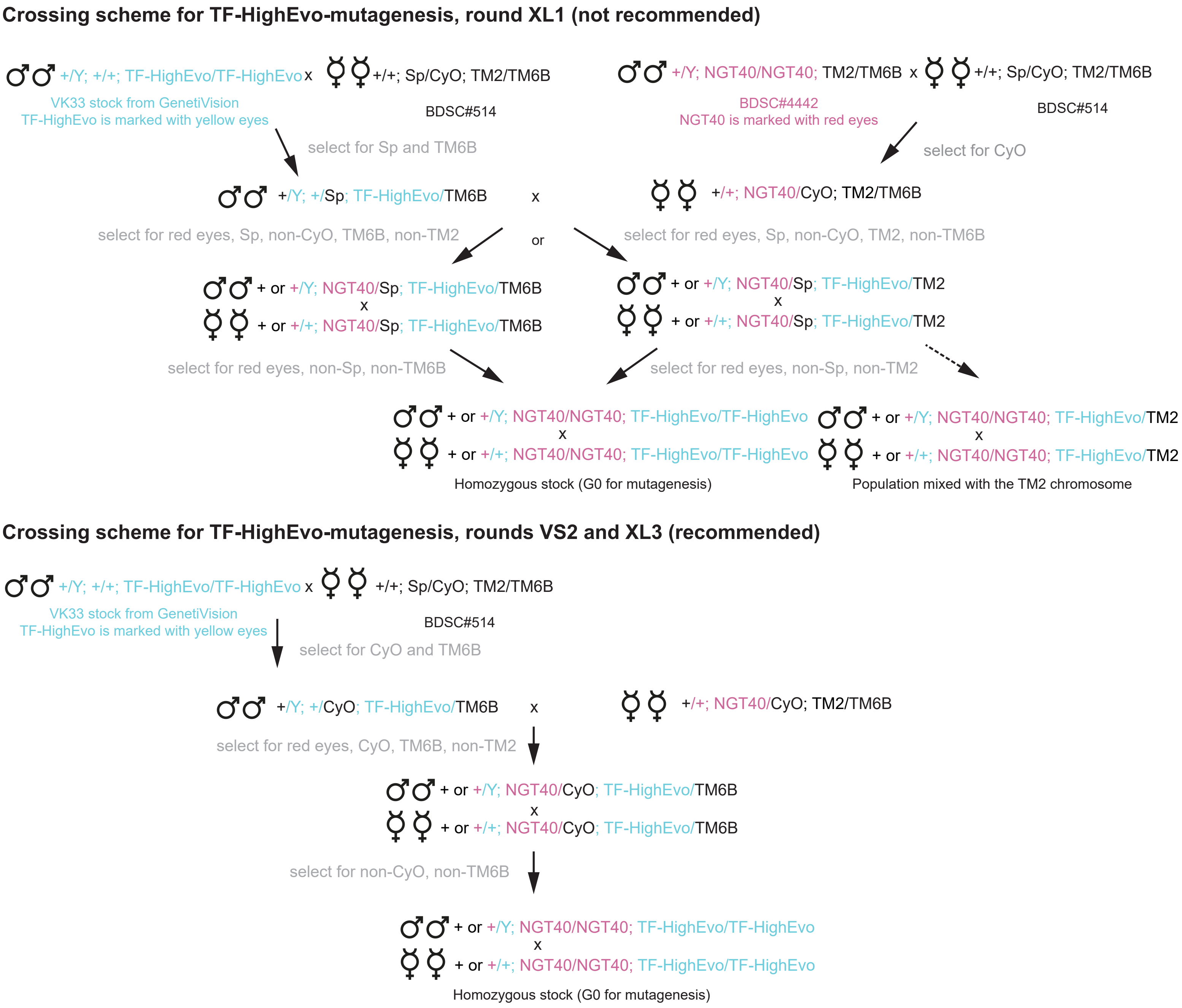

Fig. S1. Fly crosses to start TF-HighEvo-mutagenesis. NGT40 background is indicated in magenta whereas TF-HighEvo (VK33) background is indicated in cyan.

Fig. S2.

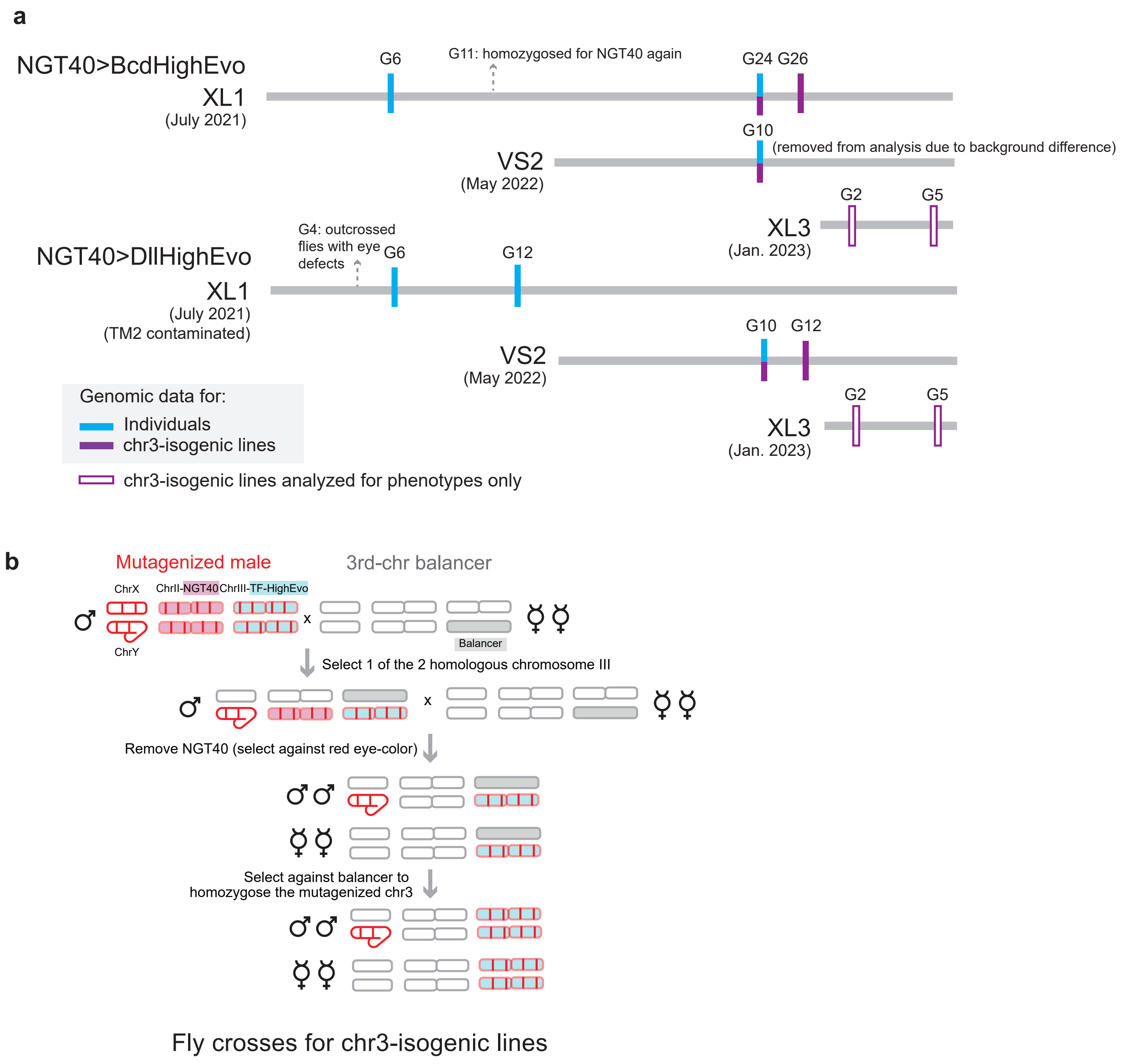

Fig. S2. Sampling scheme and isogenizing crosses. (a) Sampling scheme for genotypic and phenotypic analyses. G denotes Generation. G0 is the first generation when both NGT40 and TF-HighEvo were homozygosed. **(b)** Crossing scheme to generate chr3-isogenic lines. Mutations are represented with read lines. The balancer genotype was w;;S-b/TM6B.

Fig. S3.

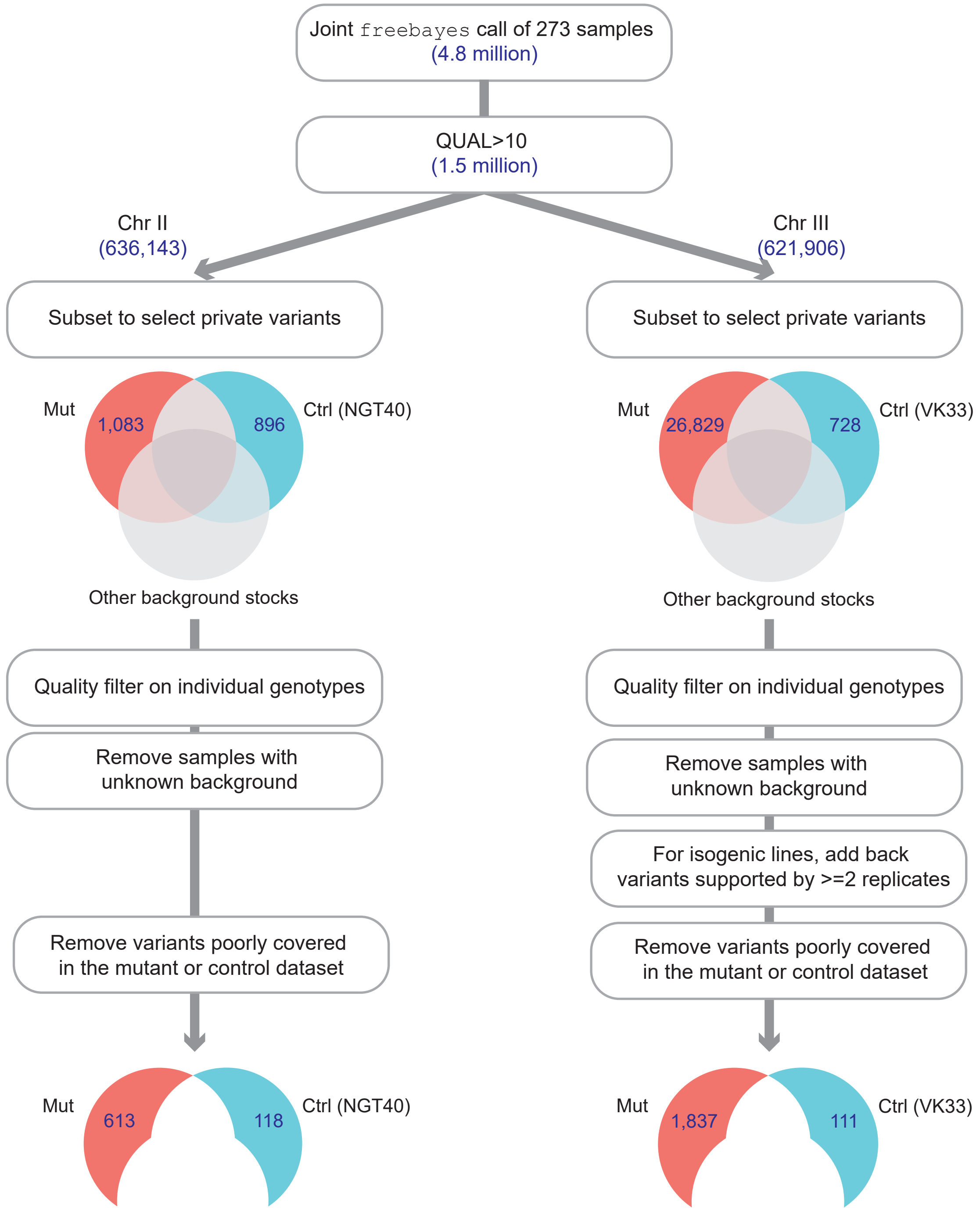

Fig. S3. Computational pipeline for identification of *de novo* mutations. The number of variants is indicated in dark blue under each step. Mut, mutagenized samples. Ctrl, control samples.

Fig. S4.

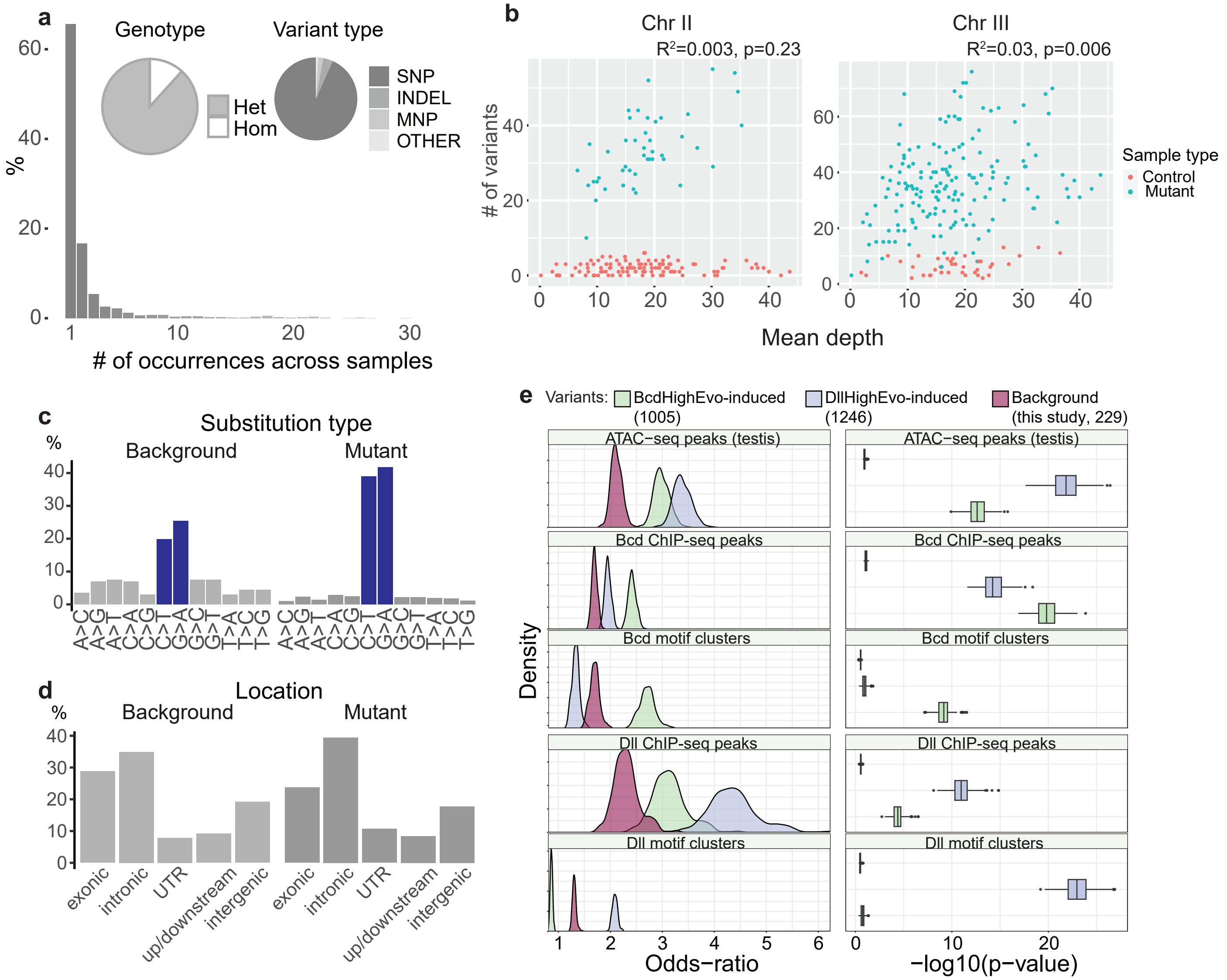

Fig. S4. Characteristics of TF-HighEvo-induced *de novo* mutations. (**a**) Distribution of allele frequency of 1,754 mutations identified across individual samples. Inserts show the distribution of heterogeneous (Het) vs. homozygous (Hom) calls (left) and the distribution of variant type (right). (**b**) Correlation between the number of private variants and mean sequencing depth per sample. P values and adjusted R squared were derived from linear models. Mutant samples include NGT40>TF-HighEvo-mutagenized samples and 29°C-mutagenized samples. (**c**) Distribution of substitution type in 2,704 variants identified in individual samples and isogenic lines. The percentage of C-to-T and G-to-A mutations (dark blue) was significantly higher in the mutagenized samples than background samples ($p < 0.001$, Fisher's exact test). (**d**) Distribution of genomic location, with no significant differences between background and mutant variant sets. (**e**) Enrichment in the overlap with regulatory sequences. C-to-T and G-to-A mutations found in BcdHighEvo-mutagenesis, DllHighEvo-mutagenesis and background stocks were compared with 10,000 randomly sampled DGRP SNPs for their likelihood to be within 100bp-distance to (1) ATAC-seq peaks in testis; (2) Bcd ChIP-seq peaks; (3) Bcd motif clusters; (4) Dll ChIP-seq peaks; and (5) Dll motif clusters. The odds ratio and p-values were derived from Fisher's exact test, with Benjamini-Hochberg correction. The distribution was generated by running the tests 500 times with different random sets of DGRP variants.

Fig. S5.

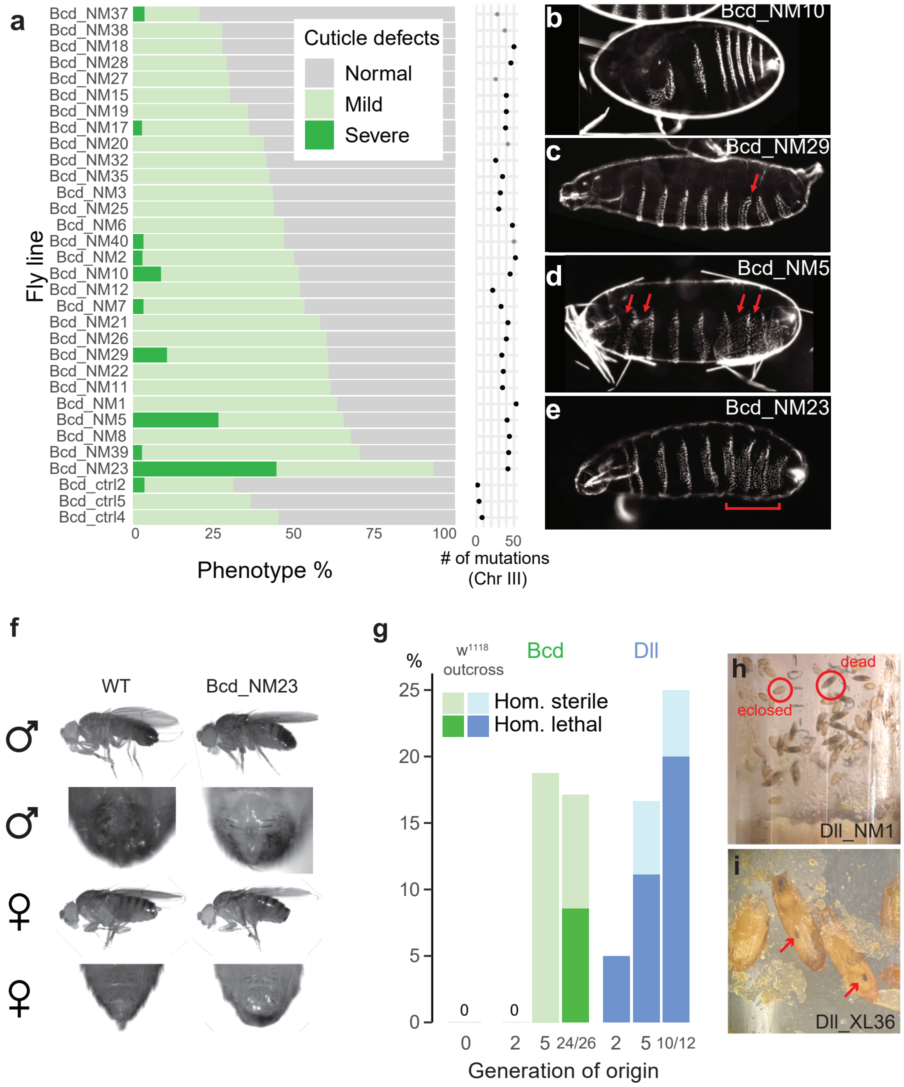

Fig. S5. Cuticle phenotypes and other developmental defects. (**a**) Percentage of cuticle phenotypes in chr3-isogenic lines derived from BcdHighEvo mutagenesis (left), ranked by the total percentage of defects within the mutant (upper) and the control (lower) group. The number of mutations in chr3 in these lines is shown on the right. (**b-e**) Severe cuticle defects found in the isogenic lines. (**f**) Genital loss of Bcd_NM23 in adult males (top two rows) and females (bottom two rows). The 2nd and the 4th rows show a close-up ventral view of the genital region. This phenotype was also found in Bcd_NM5 and Bcd_NM29. (**g**) Percentage of homozygous (Hom.)- sterile and lethal chromosomes, sampled across the time course of the mutagenesis. Generation 0 data were from the isogenizing crosses of Eye lines with mostly w1118 background (**Fig. S6c**). (**h-i**) Homozygous-lethal phenotypes at pupae stage. The heterozygous pupae with the tubby phenotype (due to the TM6B balancer) eclosed normally, indicated by empty pupae case (left circle in **h**), whereas the homozygous pupae turned black and never eclosed (right circle in **h**) in Dll_NM1. In Dll_XL36, the homozygous pupae had black melanotic tissues (red arrows in **i**) in the white-pupae stage and did not eclose.

Fig. S6.

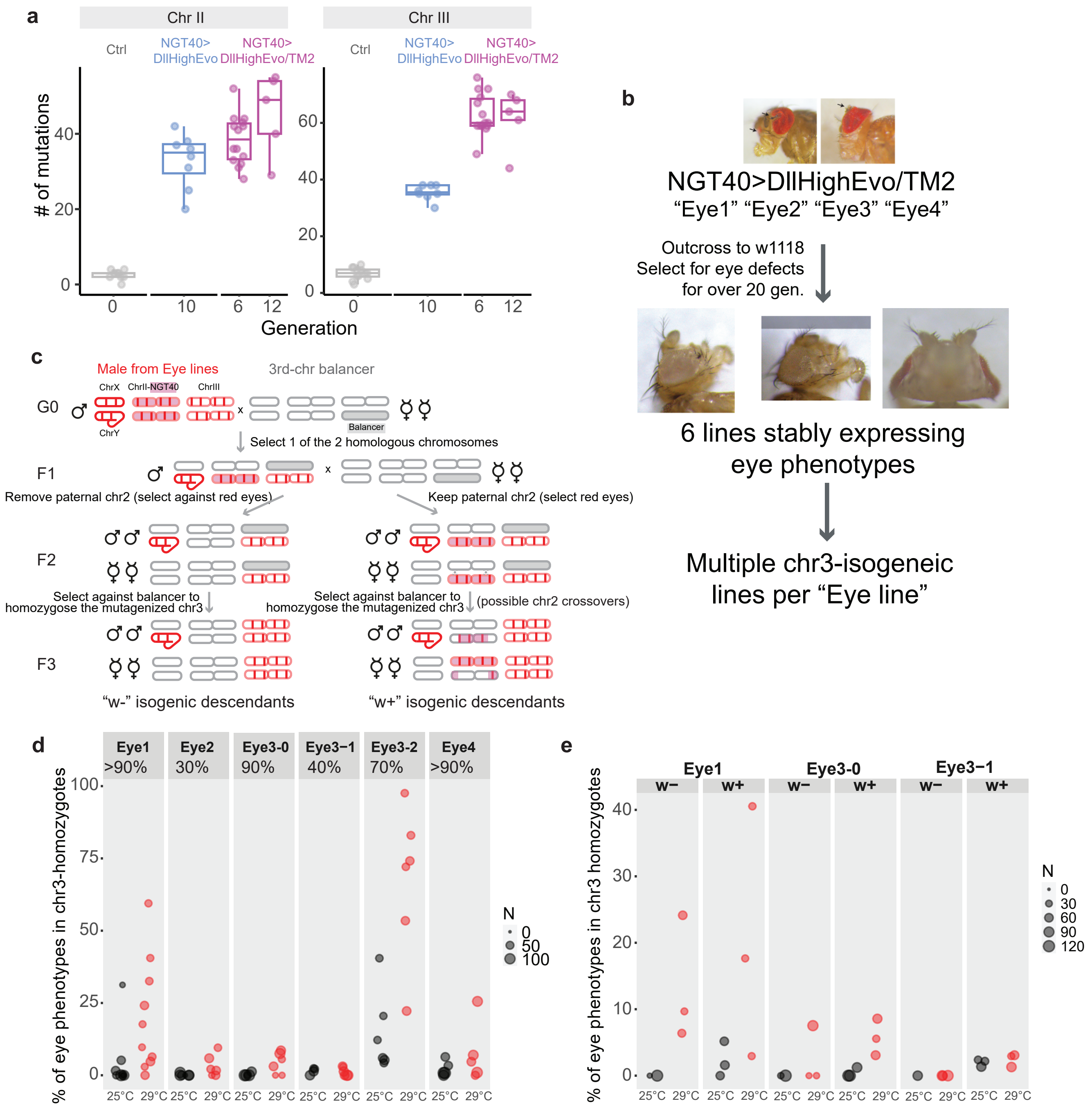

Fig. S6. Eye phenotypes and their genetic bases. (a) The number of private variants found in NGT40>DllHigh-Evo/TM2 individuals at Generation 6 and 12 was higher than those found in NGT40>DllHighEvo at Generation 10, suggesting that the presence of TM2 accelerated mutation accumulation. Only samples with a mean coverage higher than 9 were shown in this figure. **(b)** Establishing Eye lines and their chr3-isogenic descendants. **(c) C**rosses for isogenizing chr3 of flies from Eye lines. For Eye1, Eye3-0 and Eye3-1, two sets of F2 crosses were done to keep or remove the paternal chr2 based on the eye marker in the NGT40 chromosome. **(d)** Percentage of eye defects in the chr3-homozygotes, grouped by the Eye lines they originated from. The percentages below the group labels show the frequency of eye defects in the stable Eye lines. The point size reflects N, the number of individuals examined in each isogenic line for eye phenotypes. **(e)** Percentage of eye defects in the chr3-homozygotes, with comparison between w- and w+ descendants.
