# Supplementary material for: TF-High-Evolutionary: In Vivo Mutagenesis of Gene Regulatory Networks for the Study of the Genetics and Evolution of the Drosophila Regulatory Genome
Source: Mol Biol Evol. 2024 Aug 9;41(8):msae167. doi: 10.1093/molbev/msae167 (PMC11342961; doi:10.1093/molbev/msae167)
Supplement: msae167_Supplementary_Data [file msae167_supplementary_data.zip › Supp_Data_File_1.pdf]

>pXL_Bcd_AID_delN

atgtgcgcggaacccctatttgtttatttttctaaatacattcaaatatgtatccgctcatgagacaataaccctgataaatgcttcaataatattgaaaa
aggaagagtatgagtattcaacatttccgtgtcgcccttattcccttttttgcggcattttgccttcctgtttttgctcacccagaaacgctggtgaaagta
aaagatgctgaagatcagttgggtgcacgagtgggttacatcgaactggatctcaacagcggtaagatccttgagagttttcgccccgaagaacgtttt
ccaatgatgagcacttttaaagttctgctatgtggcgcggtattatcccgtattgacgccgggcaagagcaactcggtcgccgcatacactattctcaga
atgacttggttgagtactcaccagtcacagaaaagcatcttacggatggcatgacagtaagagaattatgcagtgctgccataaccatgagtgataac
actgcggccaacttacttctgacaacgatcggaggaccgaaggagctaaccgcttttttgcacaacatgggggatcatgtaactcgccttgatcgttgg
gaaccggagctgaatgaagccataccaaacgacgagcgtgacaccacgatgcctgtagcaatggcaacaacgttgcgcaaactattaactggcgaa
ctacttactctagcttcccggcaacaattaatagactggatggaggcggataaagttgcaggaccacttctgcgctcggcccttccggctggctggttta
ttgctgataaatctggagccggtgagcgtgggtctcgcggtatcattgcagcactggggccagatggtaagccctcccgtatcgtagttatctacacga
cggggagtcaggcaactatggatgaacgaaatagacagatcgctgagataggtgcctcactgattaagcattggtaactgtcagaccaagtttactca
tatatactttagattgatttaaaacttcatttttaatttaaaaggatctaggtgaagatcctttttgataatctcatgaccaaaatcccttaacgtgagtttt
cgttccactgagcgtcagaccccgtagaaaagatcaaaggatcttcttgagatcctttttttctgcgcgtaatctgctgcttgcaaacaaaaaaaccacc
gctaccagcggtggtttgtttgccggatcaagagctaccaactctttttccgaaggtaactggcttcagcagagcgcagataccaaatactgttcttcta
gtgtagccgtagttaggccaccacttcaagaactctgtagcaccgcctacatacctcgctctgctaatcctgttaccagtggctgctgccagtggcgata
agtcgtgtcttaccgggttggactcaagacgatagttaccggataaggcgcagcggtcgggctgaacggggggttcgtgcacacagcccagcttgga
gcgaacgacctacaccgaactgagatacctacagcgtgagctatgagaaagcgccacgcttcccgaagggagaaaggcggacaggtatccggtaa
gcggcagggtcggaacaggagagcgcacgagggagcttccagggggaaacgcctggtatctttatagtcctgtcgggtttcgccacctctgacttgag
cgtcgatttttgtgatgctcgtcaggggggcggagcctatggaaaaacgccagcaacgcggcctttttacggttcctggccttttgctggccttttgctca
catgttaccgtcgacgatgtaggtcacggtctcgaagccgcggtgcgggtgccagggcgtgcccttgggctccccgggcgcgtactccacctcacccat
ctggtccatcatgatgaacgggtcgaggtggcggtagttgatcccggcgaacgcgcggcgcaccgggaagccctcgccctcgaaaccgctgggcgcg
gtggtcacggtgagcacgggacgtgcgacggcgtcggcgggtgcggatacgcggggcagcgtcagcgggttctcgacggtcacggcgggcatgtcg
acaagccgaacatatgggcgcgcctagtatgtatgtaagttaataaaacccatttttgcggaaagtagataaaaaaaacatttttttttttactgcact
ggatatcattgaacttatctgatcagttttaaatttacttcgatccaagggtatttgatgtaccaggttctttcgattacctctcactcaaaatgacattcc
actcaaagtcagcgctgtttgcctccttctctgtccacagaaatatcgccgagtctttcgccgctgcgtccgctatctctttcgccaccgtttgtagcgtta
cgtagcgtcaatgtccgccttcagttgcattttgtcagcggtttcgtgacgaagctccaagcggtttacgccatcaattaaacacaaagtgctgtgccaa
aactcctctcgcttcttatttttgtttgtttttgagtgattggggtggtgattggttttgggtgggtaagcaggggaaagtgtgaaaaatcccggcaatgg
gccaagaggatcaggagctattaattcgcggaggcagcaaacacccatctgccgagcatctgaacaatgtgagtagtacatgtgcatacatcttaagt
tcacttgatctataggaactgcgattgcaacatcaaattgtatgcggcgtgagaactgcgacccacaaaaatcccaaaccgcaattgcacaaacaaat
agtgacacgaaacagattattctggtagctgttctcgctatataagacaatttttgagatcatatcatgatcaagacatctaaaggcattcattttcgact
atattctttttacaaaaaatataacaaccagatattttaagctgatcctagatgcacaaaaaataaataaaagtataaacctacttcgtaggatacttc
ggggtactttttgttcggggttagatgagcataacgcttgtagttgatatttgagatcccctatcattgcagggtgacagcggagcggcttcgcagagct
gcattaaccagggcttcgggcaggccaaaaactacggcacgctccggccacccagtccgccggaggactccggttcagggagcggccaactagccg
agaacctcacctatgcctggcacaatatggacatctttggggcggtcaatcagccgggctccggatggcggcagctggtcaaccggacacgcggact
attctgcaacgagcgacacataccggcgcccaggaaacatttgctcaagaacggtgagtttctattcgcagtcggctgatctgtgtgaaatcttaataa
agggtccaattaccaatttgaaactcagtttgcggcgtggcctatccgggcgaacttttggccgtgatgggcagttccggtgccggaaagacgaccctg
ctgaatgcccttgcctttcgatcgccgcagggcatccaagtatcgccatccgggatgcgactgctcaatggccaacctgtggacgccaaggagatgca
ggccaggtgcgcctatgtccagcaggatgacctctttatcggctccctaacggccagggaacacctgattttccaagccatggtgcggatgccacgac
atctgacctatcggcagcgagtggcccgcgtggatcaggtgatccaggagctttcgctcagcaaatgtcagcacacgatcatcggtgtgcccggcagg
gtgaaaggtctgtccggcggagaaaggaagcgtctggcattcgcctccgaggctctaaccgatccgccgcttctgatctgcgatgagcccacctccgg
actggactcctttaccgcccacagcgtcgtccaggtgctgaagaagctgtcgcagaagggcaagaccgtcatcctgaccattcatcagccgtcttccga
gctgtttgagctctttgacaagatccttctgatggccgagggcagggtagctttcttgggcactcccagcgaagccgtcgacttcttttcctagtgagttc
gatgtgtttattaagggtatctagtattacataacatctcaactcctatccagcgtgggtgcccagtgtcctaccaactacaatccggcggacttttacgt
acaggtgttggccgttgtgcccggacgggagatcgagtcccgtgatcggatcgccaagatatgcgacaattttgccattagcaaagtagcccgggata

tggagcagttgttggccaccaaaaatctggagaagccactggagcagccggagaatgggtacacctacaaggccacctggttcatgcagttccgggc
ggtcctgtggcgatcctggctgtcggtgctcaaggaaccactcctcgtaaaagtgcgacttattcagacaacggtgagtggttccagtggaaacaaat
gatataacgcttacaattcttggaaacaaattcgctagattttagatagaattgcctgattccacacccttcttagtttttttcaatgagatgtatagtttat
agttttgcagaagataaataaatttcatttaactcgcgaatattaatgagatgcgagtaacattttaatttgcagatggttgccatcttgattggcctcat
ctttttgggccaacaactcacgcaagtgggtgtgatgaatatcaacggagccatcttcctcttcctgaccaacatgacctttcaaaacgtctttgccacg
ataaatgtaagtcatgtttagaatacatttgcatttcaataatttactaactttctaatgaatcgattcgatttaggtgttcacctcagagctgccagttttt
atgagggaggcccgaagtcgactttatcgctgtgacacatactttctgggcaaaacgattgccgaattgccgctttttctcacagtgccactggtcttca
cggcgattgcctatccgatgatcggactgcgggccggagtgctgcacttcttcaactgcctggcgctggtcactctggtggccaatgtgtcaacgtcctt
cggatatctaatatcctgcgccagctcctcgacctcgatggcgctgtctgtgggtccgccggttatcataccattcctgctctttggcggcttcttcttgaa
ctcgggctcggtgccagtatacctcaaatggttgtcgtacctctcatggttccgttacgccaacgagggtctgctgattaaccaatgggcggacgtgga
gccgggcgaaattagctgcacatcgtcgaacaccacgtgccccagttcgggcaaggtcatcctggaaacgcttaacttctccgccgccgatctgccgct
ggactacgtgggtctggccattctcatcgtgagcttccgggtgctcgcatatctggctctaagacttcgggcccgacgcaaggagtagccgacatatat
ccgaaataactgcttgtttttttttttaccattattaccatcgtgtttactgtttattgcccccctcaaaaagctaatgtaattatatttgtgccaataaaaac
aagatatgacctatagaatacaagtatttccccttcgaacatccccacaagtagactttggatttgtcttctaaccaaaagacttacacacctgcatacc
ttacatcaaaaactcgtttatcgctacataaaacaccgggatatatttttatatacatacttttcaaatcgcgcgccctcttcataattcacctccaccac
accacgtttcgtagttgctctttcgctgtctcccacccgctctccgcaacacattcaccttttgttcgacgaccttggagcgactgtcgttagttccgcgcg
attcggttcgctcaaatggttccgagtggttcatttcgagtcaatagaaattagtaataaatatttgtatgtacaatttatttgctccaatatatttgtatat
atttccctcacagctatatttattctaatttaatattatgactttttaaggtaatttttttgtgacctgttcggagtgattagcgttacaatttgaactgaaagt
gacatccagtgtttgttccttgtgtagatgcatctcaaaaaaaatggtgggcataatagtgttgtttatatatatcaaaaataacaactataataataaga
atacatttaatttagaaaatgcttggatttcactggaactagggcgcgcctccggaacataatggtgcagggcgctgacttccgcgtttccagactttac
gaaacacggaaaccgaagaccattcatgttgttgctcaggtcgcagacgttttgcagcagcagtcgcttcacgttcgctcgcgtatcggtgattcattct
gctaaccagtaaggcaaccccgccagcctagccgggtcctcaacgacaggagcacgatcatgcgcacccgtggccagggccgcaagcttgcatgcct
gcaggtcggagtactgtcctccgagcggagtactgtcctccgagcggagtactgtcctccgagcggagtactgtcctccgagcggagtactgtcctccg
agcggagactctagccctagggcatgcctgcaggtcggagtactgtcctccgagcggagtactgtcctccgagcggagtactgtcctccgagcggagt
actgtcctccgagcggagtactgtcctccgagcggagactctagcgctagcgcatgcctgcaggtcggagtactgtcctccgagcggagtactgtcctc
cgagcggagtactgtcctccgagcggagtactgtcctccgagcggagtactgtcctccgagcggagactctagcactagtgcatgcctgcaggtcgga
gtactgtcctccgagcggagtactgtcctccgagcggagtactgtcctccgagcggagtactgtcctccgagcggagtactgtcctccgagcggagact
ctagcgacgtcgagcgccggagtataaatagaggcgcttcgtctacggagcgacaattcaattcaaacaagcaaagtgaacacgtcgctaagcgaa
agctaagcaaataaacaagcgcagctgaacaagctaaacaatctgcagtaaagtgcaagttaaagtgaatcaattaaaagtaaccagcaaccaagt
aaatcaactgcaactactgaaatctgccaagaagtaattattgaatacaagaagagaactctgaatagatctaaaaggtaggttcaaccactgatgc
ctaggcacaccgaaacgactaaccctaattcttatcctttacttcaggcggccgcggctcgagatggcgcaaccgccgccagatcaaaacttttaccat
catccgctgccccacacgcacacacatccgcatccgcactcccatccgcatccgcactcgcatccgcacccacatcaccaacatccgcagcttcagttg
ccgccacaattccgaaatcccttcgatttgcttttcgatgagcgaacgggagcgataaactacaactacatacgtccgtatctgcccaaccagatgccc
aagccagaggagctgcccgactctctggtgatgcggcgaccacgtcgcacccgcaccacttttaccagctctcaaatagcagagctggagcagcactt
tctgcagggacgatacctcacagccccccgacttgcggatctgtcagcgaaactagccctgggcacagcccaggtgaagatatggtttaagaaccgtc
ggcgtcgtcacaagatccaatcggatcagcacaaggaccagtcctacgaggggatgcctctctcgccgggtatgaaacagagcgatggcgatccccc
cagcttgcagactcttagcttgggtggaggagccacgcccaacgctttgactccgtcacccacgccctcaacgcccactgcacacatgacggagcact
acagcgagtcattcaacgcctactacaactacaatggaggccacaatcacgcccaggccaatcgtcacatgcacatgcagtatccttccggaggggg
gccaggacctgggtcgaccaatgtcaatggcggccagttcttccagcagcagcaggtccataatcaccagcagcaactgcaccaccagggcaaccac
gtgccgcaccagatgcagcagcagcaacagcaggctcagcagcagcaataccatcactttgacttccagcaaaagcaagccagcgcctgtcgcgtcc
tggtcaaggacgaaccggaggccgactacaacttcaacagctcgtactacatgcgatcgggaatgtctggcgccactgcatcggcatccgctgtggcc
cgaggcgctgcctcgccgggctccgaggtctacgagccattaacacccaagaatgacgaaagtccgagtctgtgtggcatcggcatcggcggacctt
gcgccatcgccgttggcgagacggaggcggccgacgacatggacgacggaacgagcaagaagacgacgctacagatcttggagcctttgaagggt
ctggacaagagctgcgacgatggcagtagcgacgacatgagcaccggaataagagccttagcaggaaccggaaatcgtggagcggcatttgccaa

atttggcaagccttcgcccccacaaggccctcagccgcccctcggaatggggggcgtggccatgggcgaatcgaaccaatatcaatgcacgatggat
acgataatgcaagcgtataatccccatcggaacgccgcgggcaactcgcagtttgcctactgcttcaattctggtggtggtctggggtcgactgacagc
ctcttgatgaaccggaggaagtttctttaccaattcaaaaatgtccgctgggctaagggtcggcgtgagacctacctgtgctacgtagtgaagaggcgt
gacagtgctacatccttttcactggactttggttatcttcgcaataagaacggctgccacgtggaattgctcttcctccgctacatctcggactgggacct
agaccctggccgctgctaccgcgtcacctggttcacctcctggagcccctgctacgactgtgcccgacatgtggccgactttctgcgagggaaccccaa
cctcagtctgaggatcttcaccgcgcgcctctacttctgtgaggaccgcaaggctgagcccgaggggctgcggcggctgcaccgcgccggggtgcaaa
tagccatcatgaccttcaaagattatttttactgctggaatactttgtagaaaaccacgaaagaactttcaaagcctgggaagggctgcatgaaaatt
cagttcgtctctccagacagcttcggcgcatccttttgcccctgtagtctagaggatctttgtgaaggaaccttacttctgtggtgtgacataattggaca
aactacctacagagatttaaagctctaaggtaaatataaaatttttaagtgtataatgtgttaaactactgattctaattgtttgtgtattttagattccaa
cctatggaactgatgaatgggagcagtggtggaatgcctttaatgaggaaaacctgttttgctcagaagaaatgccatctagtgatgatgaggctact
gctgactctcaacattctactcctccaaaaaagaagagaaaggtagaagaccccaaggactttccttcagaattgctaagttttttgagtcatgctgtgt
ttagtaatagaactcttgcttgctttgctatttacaccacaaaggaaaaagctgcactgctatacaagaaaattatggaaaaatatttgatgtatagtgc
cttgactagagatcataatcagccataccacatttgtagaggttttacttgctttaaaaaacctcccacacctccccctgaacctgaaacataaaatgaa
tgcaattgttgttgttaacttgtttattgcagcttataatggttacaaataaagcaatagcatcacaaatttcacaaataaagcatttttttcactgcattc
tagttgtggtttgtccaaactcatcaatgtatcttatcatgtctggatcgatctggccggccgtttaaacgaattcttgaagacgaaagggcctcgtgata
cgcctatttttataggttaatgtcatgataataatggtttcttagactcaggtggcacttttcggggaa

UAS binding sites (20x)

Heat shock promoter

Bicoid CDS (FBpp0081165, 1467bp, without stop codon)

linker (8 amino acids)

AID_delNES (AID without nuclear export signal)

>pXL_Dll_AID_delN

atgtgcgcggaacccctatttgtttatttttctaaatacattcaaatatgtatccgctcatgagacaataaccctgataaatgcttcaataatattgaaaa
aggaagagtatgagtattcaacatttccgtgtcgcccttattcccttttttgcggcattttgccttcctgtttttgctcacccagaaacgctggtgaaagta
aaagatgctgaagatcagttgggtgcacgagtgggttacatcgaactggatctcaacagcggtaagatccttgagagttttcgccccgaagaacgtttt
ccaatgatgagcacttttaaagttctgctatgtggcgcggtattatcccgtattgacgccgggcaagagcaactcggtcgccgcatacactattctcaga
atgacttggttgagtactcaccagtcacagaaaagcatcttacggatggcatgacagtaagagaattatgcagtgctgccataaccatgagtgataac
actgcggccaacttacttctgacaacgatcggaggaccgaaggagctaaccgcttttttgcacaacatgggggatcatgtaactcgccttgatcgttgg
gaaccggagctgaatgaagccataccaaacgacgagcgtgacaccacgatgcctgtagcaatggcaacaacgttgcgcaaactattaactggcgaa
ctacttactctagcttcccggcaacaattaatagactggatggaggcggataaagttgcaggaccacttctgcgctcggcccttccggctggctggttta
ttgctgataaatctggagccggtgagcgtgggtctcgcggtatcattgcagcactggggccagatggtaagccctcccgtatcgtagttatctacacga
cggggagtcaggcaactatggatgaacgaaatagacagatcgctgagataggtgcctcactgattaagcattggtaactgtcagaccaagtttactca
tatatactttagattgatttaaaacttcatttttaatttaaaaggatctaggtgaagatcctttttgataatctcatgaccaaaatcccttaacgtgagtttt
cgttccactgagcgtcagaccccgtagaaaagatcaaaggatcttcttgagatcctttttttctgcgcgtaatctgctgcttgcaaacaaaaaaaccacc
gctaccagcggtggtttgtttgccggatcaagagctaccaactctttttccgaaggtaactggcttcagcagagcgcagataccaaatactgttcttcta
gtgtagccgtagttaggccaccacttcaagaactctgtagcaccgcctacatacctcgctctgctaatcctgttaccagtggctgctgccagtggcgata
agtcgtgtcttaccgggttggactcaagacgatagttaccggataaggcgcagcggtcgggctgaacggggggttcgtgcacacagcccagcttgga
gcgaacgacctacaccgaactgagatacctacagcgtgagctatgagaaagcgccacgcttcccgaagggagaaaggcggacaggtatccggtaa
gcggcagggtcggaacaggagagcgcacgagggagcttccagggggaaacgcctggtatctttatagtcctgtcgggtttcgccacctctgacttgag

cgtcgatttttgtgatgctcgtcagggggggcggagcctatggaaaaacgccagcaacgcggcctttttacggttcctggccttttgctggccttttgctca
catgttaccgtcgacgatgtaggtcacggtctcgaagccgcggtgcgggtgccagggcgtgcccttgggctccccgggcgcgtactccacctcacccat
ctggtccatcatgatgaacgggtcgaggtggcggtagttgatcccggcgaacgcgcggcgcaccgggaagccctcgccctcgaaaccgctgggcgcg
gtggtcacggtgagcacgggacgtgcgacggcgtcggcgggtgcggatacgcggggcagcgtcagcgggttctcgacggtcacggcgggcatgtcg
acaagccgaacatatgggcgcgcctagtatgtatgtaagttaataaaacccatttttgcggaaagtagataaaaaaaacatttttttttttactgcact
ggatatcattgaacttatctgatcagttttaaatttacttcgatccaagggtatttgatgtaccaggttctttcgattacctctcactcaaaatgacattcc
actcaaagtcagcgctgtttgcctccttctctgtccacagaaatatcgccgagtctttcgccgctgcgtccgctatctctttcgccaccgtttgtagcgtta
cgtagcgtcaatgtccgccttcagttgcattttgtcagcggtttcgtgacgaagctccaagcggtttacgccatcaattaaacacaaagtgctgtgccaa
aactcctctcgcttcttatttttgtttgtttttgagtgattggggtggtgattggttttgggtgggtaagcaggggaaagtgtgaaaaatcccggcaatgg
gccaagaggatcaggagctattaattcgcggaggcagcaaacacccatctgccgagcatctgaacaatgtgagtagtacatgtgcatacatcttaagt
tcacttgatctataggaactgcgattgcaacatcaaattgtatgcggcgtgagaactgcgacccacaaaaatcccaaaccgcaattgcacaaacaaat
agtgacacgaaacagattattctggtagctgttctcgctatataagacaatttttgagatcatatcatgatcaagacatctaaaggcattcattttcgact
atattctttttacaaaaaatataacaaccagatattttaagctgatcctagatgcacaaaaaataaataaaagtataaacctacttcgtaggatacttc
ggggtactttttgttcggggttagatgagcataacgcttgtagttgatatttgagatcccctatcattgcagggtgacagcggagcggcttcgcagagct
gcattaaccagggcttcgggcaggccaaaaactacggcacgctccggccacccagtccgccggaggactccggttcagggagcggccaactagccg
agaacctcacctatgcctggcacaatatggacatctttggggcggtcaatcagccgggctccggatggcggcagctggtcaaccggacacgcggact
attctgcaacgagcgacacataccggcgcccaggaaacatttgctcaagaacggtgagtttctattcgcagtcggctgatctgtgtgaaatcttaataa
agggtccaattaccaatttgaaactcagtttgcggcgtggcctatccgggcgaacttttggccgtgatgggcagttccggtgccggaaagacgaccctg
ctgaatgcccttgcctttcgatcgccgcagggcatccaagtatcgccatccgggatgcgactgctcaatggccaacctgtggacgccaaggagatgca
ggccaggtgcgcctatgtccagcaggatgacctctttatcggctccctaacggccagggaacacctgattttccaagccatggtgcggatgccacgac
atctgacctatcggcagcgagtggcccgcgtggatcaggtgatccaggagctttcgctcagcaaatgtcagcacacgatcatcggtgtgcccggcagg
gtgaaaggtctgtccggcggagaaaggaagcgtctggcattcgcctccgaggctctaaccgatccgccgcttctgatctgcgatgagcccacctccgg
actggactcctttaccgcccacagcgtcgtccaggtgctgaagaagctgtcgcagaagggcaagaccgtcatcctgaccattcatcagccgtcttccga
gctgtttgagctctttgacaagatccttctgatggccgagggcagggtagctttcttgggcactcccagcgaagccgtcgacttcttttcctagtgagttc
gatgtgtttattaagggtatctagtattacataacatctcaactcctatccagcgtgggtgcccagtgtcctaccaactacaatccggcggacttttacgt
acaggtgttggccgttgtgcccggacgggagatcgagtcccgtgatcggatcgccaagatatgcgacaattttgccattagcaaagtagcccgggata
tggagcagttgttggccaccaaaaatctggagaagccactggagcagccggagaatgggtacacctacaaggccacctggttcatgcagttccgggc
ggtcctgtggcgatcctggctgtcggtgctcaaggaaccactcctcgtaaaagtgcgacttattcagacaacggtgagtggttccagtggaaacaaat
gatataacgcttacaattcttggaaacaaattcgctagattttagatagaattgcctgattccacacccttcttagtttttttcaatgagatgtatagtttat
agttttgcagaagataaataaatttcatttaactcgcgaatattaatgagatgcgagtaacattttaatttgcagatggttgccatcttgattggcctcat
ctttttgggccaacaactcacgcaagtgggtgtgatgaatatcaacggagccatcttcctcttcctgaccaacatgacctttcaaaacgtctttgccacg
ataaatgtaagtcatgtttagaatacatttgcatttcaataatttactaactttctaatgaatcgattcgatttaggtgttcacctcagagctgccagttttt
atgagggaggcccgaagtcgactttatcgctgtgacacatactttctgggcaaaacgattgccgaattgccgctttttctcacagtgccactggtcttca
cggcgattgcctatccgatgatcggactgcgggccggagtgctgcacttcttcaactgcctggcgctggtcactctggtggccaatgtgtcaacgtcctt
cggatatctaatatcctgcgccagctcctcgacctcgatggcgctgtctgtgggtccgccggttatcataccattcctgctctttggcggcttcttcttgaa
ctcgggctcggtgccagtatacctcaaatggttgtcgtacctctcatggttccgttacgccaacgagggtctgctgattaaccaatgggcggacgtgga
gccgggcgaaattagctgcacatcgtcgaacaccacgtgccccagttcgggcaaggtcatcctggaaacgcttaacttctccgccgccgatctgccgct
ggactacgtgggtctggccattctcatcgtgagcttccgggtgctcgcatatctggctctaagacttcgggcccgacgcaaggagtagccgacatatat
ccgaaataactgcttgtttttttttttaccattattaccatcgtgtttactgtttattgccccctcaaaaagctaatgtaattatatttgtgccaataaaaac
aagatatgacctatagaatacaagtatttccccttcgaacatccccacaagtagactttggatttgtcttctaaccaaaagacttacacacctgcatacc
ttacatcaaaaactcgtttatcgctacataaaacaccgggatatatttttatatacatacttttcaaatcgcgcgccctcttcataattcacctccaccac
accacgtttcgtagttgctctttcgctgtctcccacccgctctccgcaacacattcaccttttgttcgacgaccttggagcgactgtcgttagttccgcgcg
attcggttcgctcaaatggttccgagtggttcatttcgagtcaatagaaattagtaataaatatttgtatgtacaatttatttgctccaatatatttgtatat
atttccctcacagctatatttattctaatttaatattatgactttttaaggtaatttttgtgacctgttcggagtgattagcgttacaatttgaactgaaagt

gacatccagtgtttgttccttgtgtagatgcatctcaaaaaaatggtgggcataatagtgttgtttatatatatcaaaaataacaactataataataaga
atacatttaatttagaaaatgcttggatttcactggaactagggcgcgcctccggaacataatggtgcagggcgctgacttccgcgtttccagactttac
gaaacacggaaaccgaagaccattcatgttgttgctcaggtcgcagacgttttgcagcagcagtcgcttcacgttcgctcgcgtatcggtgattcattct
gctaaccagtaaggcaaccccgccagcctagccgggtcctcaacgacaggagcacgatcatgcgcacccgtggccagggccgcaagcttgcatgcct
gcaggtcggagtactgtcctccgagcggagtactgtcctccgagcggagtactgtcctccgagcggagtactgtcctccgagcggagtactgtcctccg
agcggagactctagccctagggcatgcctgcaggtcggagtactgtcctccgagcggagtactgtcctccgagcggagtactgtcctccgagcggagt
actgtcctccgagcggagtactgtcctccgagcggagactctagcgctagcgcatgcctgcaggtcggagtactgtcctccgagcggagtactgtcctc
cgagcggagtactgtcctccgagcggagtactgtcctccgagcggagtactgtcctccgagcggagactctagcactagtgcatgcctgcaggtcgga
gtactgtcctccgagcggagtactgtcctccgagcggagtactgtcctccgagcggagtactgtcctccgagcggagtactgtcctccgagcggagact
ctagcgacgtcgagcgccggagtataaatagaggcgcttcgtctacggagcgacaattcaattcaaacaagcaaagtgaacacgtcgctaagcgaa
agctaagcaaataaacaagcgcagctgaacaagctaaacaatctgcagtaaagtgcaagttaaagtgaatcaattaaaagtaaccagcaaccaagt
aaatcaactgcaactactgaaatctgccaagaagtaattattgaatacaagaagagaactctgaatagatctaaaaggtaggttcaaccactgatgc
ctaggcacaccgaaacgactaaccctaattcttatcctttacttcaggcggccgcggctcgagATGGACGCCCCCGACGCCCCGCACACG
CCCAAGTACATGGACGGCGGGAACACGGCGGCCAGCGTCACGCCCGGCATTAACATCCCGGGAAAGTCCGCCTTC
GTGGAGCTGCAGCAGCACGCCGCCGCCGGGTACGGCGGCATCCGGAGCACCTATCAGCATTTCGGACCGCAGGG
CGGCCAGGATTCCGGCTTCCCCAGTCCGCGCAGCGCCCTCGGCTACCCGTTCCCGCCAATGCACCAGAACTCCTAC
TCCGGCTACCATCTGGGCAGCTACGCCCCGCCCTGCGCCAGTCCACCGAAGGATGACTTCTCCATCTCCGATAAGT
GCGAGGACTCCGGCCTCCGCGTCAACGGCAAGGGCAAGAAGATGCGCAAGCCCCGCACCATCTACAGCTCGCTGC
AACTGCAGCAGCTCAACCGCCGCTTCCAGCGTACCCAATACCTGGCCCTGCCGGAGCGAGCGGAGCTGGCGGCGA
GTCTGGGCCTCACGCAAACGCAGGTGAAAATCTGGTTCCAGAACCGACGCTCCAAGTACAAAAAGATGATGAAGG
CGGCCCAGGGCCCGGGCACCAACAGCGGCATGCCTCTGGGCGGGGGAGGACCCAATCCCGGCCAGCATAGCCCC
AATCAAATGCACAGTGGCGGTAATAACGGCGGTGGCTCGAACAGCGGCTCACCCTCACATTATCTACCTCCCGGA
CACAGTCCAACGCCATCGAGTACACCCGTGTCCGAGCTTTCACCTGAATTCCCACCAACGGGCCTCAGTCCGCCGA
CGCAAGCGCCGTGGGACCAGAAACCGCACTGGATCGACCACAAGCCGCCGCCCCAAATGACGCCACAACCACCTC
ATCCAGCGGCAACGCTACATCCGCAGACGCACCACCACAACCCGCCGCCCCAGATGGGTGGCTATGTGCCCCAGT
ACTGGTACCAGCCCGAGACGAATCCATCACTAGTGACTGTTTGGCCGGCGGTCtctggtggtggtctggggtcgactgacagc
ctcttgatgaaccggaggaagtttctttaccaattcaaaaatgtccgctgggctaagggtcggcgtgagacctacctgtgctacgtagtgaagaggcgt
gacagtgctacatcctttttcactggactttggttatcttcgcaataagaacggctgccacgtggaattgctcttcctccgctacatctcggactgggacct
agaccctggccgctgctaccgcgtcacctggttcacctcctggagcccctgctacgactgtgcccgacatgtggccgactttctgcgagggaaccccaa
cctcagtctgaggatcttcaccgcgcgcctctacttctgtgaggaccgcaaggctgagcccgaggggctgcggcggctgcaccgcgccggggtgcaaa
tagccatcatgaccttcaaagattatttttactgctggaatacttttgtagaaaaccacgaaagaactttcaaagcctgggaagggctgcatgaaaatt
cagttcgtctctccagacagcttcggcgcatccttttgcccctgtagtctagaggatctttgtgaaggaaccttacttctgtggtgtgacataattggaca
aactacctacagagatttaaagctctaaggtaaatataaaatttttaagtgtataatgtgttaaactactgattctaattgtttgtgtattttagattccaa
cctatggaactgatgaatgggagcagtggtggaatgcctttaatgaggaaaacctgttttgctcagaagaaatgccatctagtgatgatgaggctact
gctgactctcaacattctactcctccaaaaaagaagagaaaggtagaagaccccaaggactttccttcagaattgctaagttttttgagtcatgctgtgt
ttagtaatagaactcttgcttgctttgctatttacaccacaaaggaaaaagctgcactgctatacaagaaaattatggaaaaatatttgatgtatagtgc
cttgactagagatcataatcagccataccacatttgtagaggttttacttgctttaaaaaacctcccacacctccccctgaacctgaaacataaaatgaa
tgcaattgttgttgttaacttgtttattgcagcttataatggttacaaataaagcaatagcatcacaaatttcacaaataaagcatttttttcactgcattc
tagttgtggtttgtccaaactcatcaatgtatcttatcatgtctggatcgatctggccggccgtttaaacgaattcttgaagacgaaagggcctcgtgata
cgcctatttttataggttaatgtcatgataataatggtttcttagactcaggtggcacttttcggggaa

UAS binding sites (20x)

Heat shock promoter

Distal-less CDS (FBpp0072286, 981bp, without stop codon)

linker (8 amino acids)

AID_delNES (AID without nuclear export signal)
